# Landscape Genomic Tools Can Inform Future Rootstock and Farmland Selection for an Agricultural Tree Nut From Its Wild Relatives

**DOI:** 10.1111/mec.70420

**Published:** 2026-06-12

**Authors:** Ryan C. Buck, Diego J. Zapata, Victoria L. Sork

**Affiliations:** ^1^ Department of Ecology and Evolutionary Biology University of California Los Angeles California USA; ^2^ La Kretz Center for California Conservation Sciences University of California Los Angeles California Los Angeles USA; ^3^ Institute of the Environment and Sustainability University of California Los Angeles California USA

**Keywords:** adaptedness, agricultural landscape genomics, black walnuts, climate change, crop wild relative, rootstock sourcing

## Abstract

Climate change is threatening crop yield of a broad range of agricultural species, impacting global food security and trade. Crop wild relatives may contain climate adaptations that can be quickly introduced into cultivars, especially in perennial tree crops that use rootstock. Identifying climate resilient genotypes that can potentially be used as alternative rootstock is imperative to mitigate the impacts of climate change. Here, we used whole genome sequence data of 59 wild *Juglans hindsii* (Northern California black walnut) and 39 wild *J. californica* (Southern California black walnut) adult trees to: (i) determine predicted adaptedness to future climate based on landscape genomic models, (ii) explore their adaptedness if used as rootstock in existing walnut orchards and (iii) identify potential future planting sites within existing croplands. Wild *J. hindsii* has the highest predicted adaptedness to the future climate of Northern and Central California walnut orchards, while wild *J. californica* has the highest predicted adaptedness to Southern and Central California walnut orchards. *Juglans californica* has high adaptedness to the future climate of more existing cropland than *J. hindsii* does. If walnut farmers wanted to test new rootstock sources for their existing orchards or convert their farmland into walnut orchards, this study informs the exploration of such ideas. We illustrate how landscape genomic tools can be utilized in agricultural contexts as first steps in identifying climate adapted genotypes.

## 1 Introduction

Climate change is threatening the yield of crop species globally (Challinor et al. 2014; Rezaei et al. 2023), affecting the stability of food production and reliant economies. In California, the top agricultural state in the United States (California Department of Food and Agriculture 2024) and a significant contributor to the global crop market (Carter 2020), changes in temperature, precipitation and climate extremes are challenging the state's large agricultural sector, especially in water availability (Pathak et al. 2018; Parker et al. 2020). Agriculture accounts for 40% of California's water demand (Peterson et al. 2023), with nearly 90% of harvested crops using irrigation (Pathak et al. 2018), a practice that farmers will have to increasingly rely on to mitigate the impacts of heat stress. The already mounting water scarcity issues and overdrafting of groundwater in California (Famiglietti et al. 2011; Cook et al. 2015; Richey et al. 2015) make the projected decrease in water availability a significant threat to crop yield and farmable land (Tanaka et al. 2006). Identifying climate‐resilient genotypes that demand less water is essential for buffering crop yield against the impacts of climate change.

Permanent crops, one of the most profitable commodities in California, are especially vulnerable to the impacts of climate change (Pathak et al. 2018) because they are grown for decades and will likely experience drastically different climate conditions than those present at establishment. Compared to annual crops, permanent crops have longer generation times that limit the rate of selection for adaptive traits (Jump and Peñuelas 2005). As a result, they will likely need to rely on standing genetic variation present either in existing cultivars or sourced from crop wild relatives. Crop wild relatives, the non‐domesticated species closely related to or even the progenitors of cultivated plants (Choudhary et al. 2017), typically have more genetic diversity due to their larger effective population sizes, broader environmental exposure, and lack of artificial selection history (Olsen and Gross 2008; Zhang et al. 2017). Further, wild relatives likely contain genetic variation shaped by adaptation to their local environments and thus represent critical reservoirs of climate‐adapted diversity for crop improvement (Maxted and Kell 2009; Zhang et al. 2017; Dempewolf et al. 2017). Crop wild relatives have been used to introduce genetic diversity and adaptive traits, such as disease resistance and drought tolerance, in numerous species (Escalant et al. 2002; Ceccarelli et al. 2004; Yadav et al. 2004; Hajjar and Hodgkin 2007; Choudhary et al. 2017; Migicovsky and Myles 2017; Kapazoglou et al. 2023). In crop trees, the opportunity to introduce diversity from wild relatives is amplified by the widespread use of grafted rootstocks (Warschefsky et al. 2016), a process in which the fruit‐bearing shoot (scion) of one plant is fused onto the root system (rootstock) of another. Rootstocks can provide instant physiological benefits to their grafted scions such as disease resistance, abiotic tolerance, and increased yield (Koepke and Dhingra 2013), all without genetic changes to the scion. Using crop wild relatives as rootstock for permanent crops could allow for the immediate introduction of climate‐adapted diversity and traits without years of breeding experiments or the disruption of established cultivars.

Some benefits of using crop wild relatives in rootstock are apparent in the crop tree, walnuts, which are grown almost exclusively on rootstock in California (California Walnut Board 2025). The main walnut crop grown is the domesticated *Juglans regia* (Persian walnut), which is grafted onto rootstock of either wild *J. hindsii* (Northern California black walnut) or, more commonly, the *Paradox* hybrid created from crossing *J. regia* and *J. hindsii* . Trees with the Paradox rootstock are more vigorous, produce higher yield (Grant and McGranahan 2006; Grant 2015), have less dieback (Hasey et al. 2004), and are less susceptible to pests and disease like nematodes (*Pratylenchus vulnus*; Hasey et al. 2004) and root and crown rot (caused by *Phytophtora*; Mircetich and Matheron 1983), but may produce lower nut quality (Connell et al. 2010). An additional wild black walnut species, *J. californica* (Southern California black walnut), exists in Southern California and is closely related to *J. hindsii* . No studies have been done examining its use as potential rootstock, but its inhabitation of the hotter, drier environments present in Southern California and its predicted increase in distribution in most future climate models (Riordan et al. 2015; Rose et al. 2023) may suggest it has adaptations suitable for future climates. To date, the purpose of developing walnut rootstock has largely been to avoid pests and disease (Matheron and Mircetich 1985; McKenna and Epstein 2003; Buzo et al. 2009; Baumgartner et al. 2013) but none have explored the potential of developing rootstock to mitigate future climate change. Yet, walnut is a very climate‐sensitive crop tree (Gauthier and Jacobs 2011; Pathak et al. 2018), with projected yield reductions of around 10% by 2050 (Lobell et al. 2006). California alone produces 99% of US walnuts (Vuppalapati 2023) and half of the global trade supply (California Walnut Board 2025), meaning that any reduction in yield would present a significant challenge to farmers and global supply. Investigating walnut wild relatives for rootstock that are preadapted to future climate may be a solution to curb projected walnut declines without replacing the established fruiting cultivar.

A powerful approach for identifying climate‐adapted genotypes is the use of landscape genomic tools. Landscape genomics examines how genomic variation is associated with environmental gradients (Sork et al. 2013). These genotype‐environment analyses (GEAs) are typically used in conservation and evolutionary biology research to study local adaptation and select climate‐adapted seed sources (e.g., Gougherty et al. 2021; Mead et al. 2024; Buck et al. 2026a, 2026b), but can effectively be applied to agricultural practices (Campbell et al. 2025). Because these methods assume local adaptation, GEA‐based approaches are more applicable to crop wild relatives than modern cultivars, which have experienced artificial selection that can obscure signals of local environmental adaptation (Campbell et al. 2025). Recent agricultural landscape genomic studies have begun to identify putatively adaptive loci in crop wild relatives that can then be used in the selection of climate‐resilient genotypes and be incorporated into breeding programs (e.g., Neyhart et al. 2022; Campbell et al. 2024; Halpin‐McCormick et al. 2025). Here, we demonstrate how these landscape genomic tools can be extended even further by identifying wild seed or rootstock sources that may be preadapted to future climate conditions as well as farmland whose future climates may match the adaptive environment of crop wild relatives.

Our overarching goal is to illustrate how landscape genomic tools can be utilized in agricultural practices for a tree crop species to find climate‐resilient solutions. Our specific objectives are to (i) assess the adaptedness of two walnut crop wild relatives, *J. hindsii* and *J. californica* , to future climate, (ii) identify stands of wild *J. hindsii* or *J. californica* that could be tested as potential rootstock or hybrid rootstock parents by determining which stands are more preadapted to the future climate of existing walnut orchards and (iii) identify existing croplands that could be converted to walnut orchards by determining which croplands will have future climates to which wild *J. hindsii* or *J. californica* are preadapted. Our findings inform new approaches to the cultivation of this crop tree and identify potential limitations that would need further investigation.

## 2 Materials and Methods

### 2.1 Study Species

**FIGURE 1:**
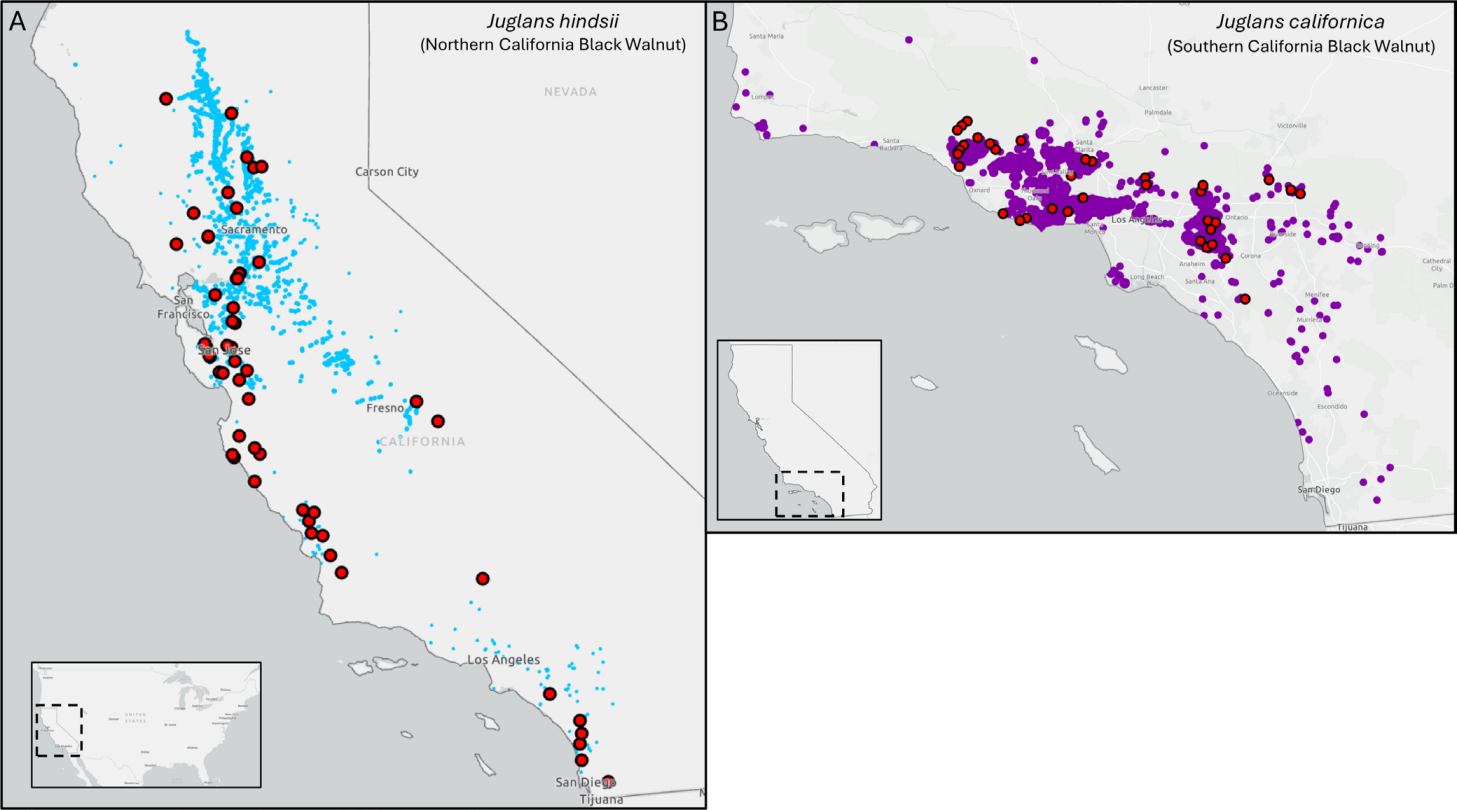
Ranges of (A) *J. hindsii* (Northern California black walnut) in blue and (B) *J. californica* (Southern California black walnut) in purple, with their final datasets' sampling localities overlaid as red dots. Note that ranges are buffered for ease of visualization and are likely smaller than pictured.

Walnut is the oldest tree food known to man (Vuppalapati 2023). Two native endemic black walnut species exist in California, *J. hindsii* (Northern California black walnut) and *J. californica* (Southern California black walnut), serving as foundation species that structurally and functionally support the walnut woodland ecosystem they comprise. In addition, they are culturally significant and were widely used by Indigenous Peoples of California for food, dye, and games (Timbrook and Chapman 2007; Wilken 2018). The two species inhabit different ranges, with Northern California black walnut distributed throughout the Central Valley from Redding to Fresno and along the coast from San Francisco to San Diego (Figure 1A). Southern California black walnut is restricted to the coast of Southern California, distributed from Santa Barbara to San Diego (Figure 1B). Both species are winter‐deciduous hardwood trees that occur along riparian corridors with high water‐holding capacity (Keeley 1990; Anderson 2002). Historically, the species have been hard to distinguish, resulting in some taxonomic controversy (Anderson 2002); however, recent studies have proposed differences in leaf morphology that can be used to delineate the taxa (Baldwin and Goldman 2012; Zapata 2024). *Juglans californica* is listed as Near Threatened on the IUCN Red List (Stritch and Barstow 2019), having already lost 31% of its suitable habitat to urbanization alone, with predicted future losses of up to 60% (Riordan et al. 2015).

### 2.2 Sample Collection and Range Extrapolation

In August of 2020 through July of 2021 and in November of 2023, leaf samples from 59 *J. hindsii* and 57 *J. californica* individuals were collected across their respective ranges (Figure 1A,B and Figure S1). This individual‐based sampling design aimed to capture the environmental and genetic variation across the ranges of each wild walnut relative (Figures S2 and S3). Multiple leaves were collected from each tree and immediately placed on ice before being transferred to a −80°C freezer for future analyses and long‐term storage.

The current ranges of *J. hindsii* and *J. californica* were assembled using occurrence data from CALVEG (USDA Forest Service 2018), VegCAMP (California Department of Fish and Wildlife 2024) and Consortium of California Herbaria (CCH2). Each concatenated species range was then expanded with a 2.5 km buffer to aid in visualization of results so actual ranges are likely thinner than pictured here. The current ranges of California walnut orchards and all cropland types were obtained from the USDA Cropland Data Layer (USDA National Agricultural Statistics Service Cropland Data Layer 2023). All geofiles were mapped and merged in ArcGIS Pro (ESRI 2025).

### 2.3 DNA Extraction and Sequencing

Approximately 50 mg of leaf tissue from each sample was flash‐frozen in liquid nitrogen before bead grinding. DNA was then extracted using a modified version of the Qiagen DNeasy Plant Mini Kit protocol preceded by a prewash step to remove polyphenols, following Mead et al. (2024) and Li et al. (2007). The prewash buffer, consisting of 100 μL of Tris, 100 μL of ethylenediaminetetraacetic acid (EDTA), 200 μL of 5 M NaCl, 600 μL of molecular grade water, and 0.01 g of polyvinylpyrrolidone (PVP), was applied twice to each sample. Extracted DNA was then sent to UC Davis DNA Technologies and Expression Analysis Core Laboratory for library preparation using a custom seqWell kit and whole‐genome sequencing (WGS) on a NovaSeq 6000 150 bp paired‐end sequencer.

### 2.4 Filter and Variant Calling

To produce a whole‐genome dataset, adapter sequences were trimmed from raw reads using Trim Galore (https://github.com/FelixKrueger/TrimGalore), removing reads less than 20 bp in length to exclude low‐quality fragments. Reads were then aligned to the *J. hindsii* reference genome (NCBI assembly GCA_041380795.1), using BWA‐MEM v07.17 (Li 2013) with ‘markShorterSplits’ (to improve handling of split alignments) and ‘readGroupHeaderLine’ (to assign sample metadata) options enabled. Duplicate reads were marked and removed using the MarkDuplicates tool in GATK v4.2.0.0 (Van der Auwera and O'Connor 2020). Variants were called using the ‘HaplotypeCaller’ tool in GATK with the ‘emit‐ref‐confidence’ option set to ‘GVCF’. GVCFs were then imported into GenomicsDB via GATK GenomicsDBImport and genotyped using ‘GenotypeGVCFs’. Variants were hard‐filtered to remove low‐confidence calls using the ‘VariantFiltration’ tool in GATK, with single nucleotide polymorphisms (SNPs) and indels filtered separately. For SNPs, variants with quality by depth (QD) < 2, quality (QUAL) < 30, mapping quality (MQ) < 40, phred‐scaled strand bias (FS) > 60, symmetric odds ratio strand bias (SOR) > 3, mapping quality rank sum (MQRankSum) < −12.5, and read position rank sum (ReadPosRankSum) < −8 were removed (Mead et al. 2024; Buck et al. 2025, 2026a, 2026b). Indels with QD < 2, FS > 200, QUAL < 30, and ReadPosRankSum < −20 were removed (Mead et al. 2024; Buck et al. 2025, 2026a, 2026b). Repetitive regions of the genome were identified through RepeatMasker v4.1.2 (Smit et al. 2015) using the available *Quercus*‐derived repeat library as a proxy due to the lack of a *Juglans*‐specific repeat database and their relatively close phylogenetic relationship (order Fagales). Repetitive regions were then removed using vcftools v0.1.16 (Danecek et al. 2011) to exclude potentially misaligned loci. Species were subsequently split into two datasets based on preliminary genetic structure analyses that indicated clear species‐level differentiation (Figure S4) and 18 admixed individuals were removed for a final total of 59 WGS samples of *J. hindsii* and 39 of *J. californica* . Using vcftools, only biallelic SNPs were selected, individual genotypes with depth < 5 were set to missing, and SNPs with a mean depth across samples < 5, with a minor allele frequency < 0.01, and with ≥ 10% missingness across individuals were removed. The resulting filtered VCF files were converted to BED file format using PLINK v1.90b6.24 (Chang et al. 2015), and variants were pruned for linkage disequilibrium separately for each species using a window size of 50 variants, a window shift value of 10, and an R^2^ threshold of 0.1.

Genetic structure analyses were separately run on these two filtered and LD‐pruned datasets: one of 59 *J. hindsii* individuals with 100,528 SNPs and one of 39 *J. californica* individuals with 715,275 SNPs. The discrepancy in number of SNPs between the two datasets is expected given both species were aligned to the *J. hindsii* reference genome, so the SNPs identified in the *J. californica* dataset represent not only intraspecific variation but also interspecific divergence from the reference. For analyses requiring no missing data, SNPs were imputed by assigning missing genotypes the most common allele at that locus (total missingness was < 7% in the *J. hindsii* filtered dataset and < 3% in the *J. californica* filtered dataset).

### 2.5 Environmental Data

Ten baseline and future soil, terrain, and hydroclimatic variables (Table S1) identified as important to the California Floristic Provence by Rose et al. (2023) were extracted for each locality. Hydroclimatic variables from the Basin Characterization Model version 8 (BCM) (Flint et al. 2021; Stern et al. 2024) included actual evapotranspiration (AET), climatic water deficit (CWD), winter precipitation (PPT_djf), summer precipitation (PPT_jja), and minimum monthly temperature (TMN). Soil variables, including available water capacity (AWC), soil depth (Depth), percent clay (PCT_clay), and soil pH (PH), were acquired from the gridded National Soil Survey Geographic Database (gNATSGO, Soil Survey Staff 2020). Finally, 15 landform types (Terrain) were included that reflect physiographic diversity and were developed for climate adaptive resource management (Theobald et al. 2015). Terrain and soil variables were resampled to match the 270 m spatial resolution of the hydroclimatic data, using nearest neighbour resampling for the categorical landform data and bilinear resampling for the continuous soil variables. Variables from the 30‐year averages of 1981–2010 (referred to as ‘baseline’) were selected to represent historical conditions closest to what the sampled mature trees grew in, noting that most sampled trees were likely older than 10 to 40 years of age. Future hydroclimatic variables were extracted from two climate models (CNRM‐CM5: warm‐wet, HadGEM2‐ES: hot‐dry) among those recommended by California's Fourth Climate Change Assessment (Pierce et al. 2018), two representative concentration pathways (RCPs) (4.5: emission reduction, 8.5: emission increase), and 30‐year averages of two future time periods (2040–2069 and 2070–2099). Landform and soil conditions were assumed to remain constant through time and were used to project models for both baseline and future climate conditions.

### 2.6 Genetic Structure and Variation

As background, the underlying genetic structure of *J. hindsii* and *J. californica* was separately quantified across each of their ranges. First, to assess the distribution of genetic variation, a Principal Components Analysis (PCA) was implemented in the R package *vegan* (Oksanen et al. 2024). Next, the maximum likelihood program ADMIXTURE (Alexander et al. 2009), was conducted to estimate discrete genetic clusters (*K*) within the range and calculate ancestry coefficients, testing *K* values 1–10 and using the lowest cross‐validation value to select the best *K*. Ancestry coefficients (*Q* values) were visualized using pophelper (Francis 2017). Finally, to test if genetic gradients are a result of isolation by distance or isolation by environment, we used partial Mantel tests (Mantel 1967) in *vegan* where genetic distance was calculated as the Euclidean distance between genotypes, geographic distance was calculated as Haversine distances using the ‘distHaversine’ command in the *geosphere* package (Hijmans 2024), and environmental distance was calculated as the Euclidean distance among individuals based on *z*‐score scaled environmental variables extracted at each individual's location.

### 2.7 Genotype‐Environment Associations and Climate Adaptedness

To examine the relationship between genetic variation and environmental gradients and to identify candidate adaptive SNPs associated with environmental gradients, a redundancy analysis (RDA) was performed with the set of 10 environmental variables from Rose et al. (2023) described above. RDA, a multivariate genotype‐environment association method, accounts for variation from all environmental variables simultaneously, resulting in higher true positive rates than other univariate and multivariate approaches (Forester et al. 2018). Partial redundancy analyses (pRDAs) separately and jointly evaluated the contributions of climate/soil, geography, and population structure on genetic variation in the R package *vegan*, with an ANOVA summarizing each factor's influence via the ‘anova’ function in R (Tables S2 and S3; Capblancq and Forester 2021). Six samples were removed from the *J. hindsii* analyses due to missing raster data. A partial, constrained RDA analysis was then performed using the 10 environmental variables conditioned on the geographic variables (latitude, longitude, latitude × longitude), and population structure (PC1) to identify outlier loci, retaining two constrained RDA axes. The Mahalanobis distances were calculated, corrected for inflation factor, and transformed into *p* values using a chi‐squared distribution with *K* = 2 degrees of freedom (François et al. 2016; Luu et al. 2017; Capblancq et al. 2018). We identified outliers with *p* values smaller than the conservative Bonferroni‐corrected significance threshold of α = 0.01/*n* (where *n* is the number of loci tested) and retained the top 10% of significant outliers as climate‐associated SNPs (141 in *J. hindsii* and 1083 in *J. californica* ) that were then used in the gene ontology and adaptedness analyses (Figures S5 and S6; Capblancq et al. 2020; Capblancq and Forester 2021). The potential function associated with each climate‐associated SNP was then examined by utilizing the well‐annotated *J. regia* reference genome (Walnut2.0, Marrano et al. 2020) (the *J. hindsii* genome is not annotated). Annotations from the *J. regia* reference genome were lifted to the *J. hindsii* genome using Liftoff (Shumate and Salzberg 2021) and known genes within ±1kbp of each SNP were identified as preliminarily functional.

To assess the current climate‐associated genetic variation across Northern and Southern California walnuts' ranges and predict the mismatch between future environmental variables and current climate‐associated genetic variation, Gradient Forest (GF) analyses were implemented separately for each species on their respective climate‐associated loci using the 10 environmental variables. Gradient Forest is a machine learning random forest technique that models multivariate allele frequencies as ensembled nonlinear regression functions of environmental variables (Ellis et al. 2012; Fitzpatrick et al. 2021). We chose to use GF due to its ability to handle polygenic loci and multivariate environmental data, resulting in better model performance than other methods (Rellstab et al. 2021; Lind and Lotterhos 2024). These analyses are built on the genotypes of sampled individuals, but the climate associations and resulting predictions are limited to the resolution of climate data. Adaptedness is therefore predicted for entire grid cells and, as such, we refer to the trees existing in those grid cells as ‘stands’ because they are likely composed of multiple individuals. The ‘gradientForest’ function was run with the number of trees set to 500, correlation threshold to 0.5 and max level set to log_2_(0.368 × number of individuals/2) (Ellis et al. 2012). Adaptive genomic turnover was then predicted for the whole range using the ‘predict’ function from the *gradientForest* R package and mapped using Fitzpatrick et al. (2021) function ‘pcaToRaster’. Adaptedness between the current climate‐associated genetic variation and each RCP and time period were calculated using a modified version of Fitzpatrick et al. (2021) code which takes the standard deviation of the current and future GF predictions and then multiplies by −1 to produce climate adaptedness values. The resulting adaptedness values were then mapped in ArcGIS Pro (ESRI, Redlands, CA).

### 2.8 Future Rootstock Sourcing and Growing Site Analyses

To identify wild walnut stands that may serve as rootstock sources preadapted to the future climate of existing walnut orchards in California (Figure S7), the predicted adaptedness of potential rootstock sources following hypothetical transfer to future locations was calculated for each species independently. Adaptedness values were calculated between each donor cell (raster grid cell within the current range of each species representing a hypothetical rootstock source) and each recipient cell (raster grid cell within the existing walnut orchards representing a hypothetical planting location under future climate) for each climate scenario. For this exploratory study, we examine sections of California because no specific rootstock source or planting site have been identified as candidate rootstock or cropland for climate‐focused improvement. Thus, to assess the general utility of the landscape genomic approach and exemplify how adaptedness might differ among planting sites, we break existing walnut orchards in California into geographic sections. Because California spans vast geographic and ecological regions, the state was broken into thirds by latitude, with Northern (42° to 38.87°N, mean climate water deficit = 571 mm), Central (38.87° to 35.57°N, mean CWD = 934 mm), and Southern (35.57° to 32.60°N, mean CWD = 1135 mm) sections to determine whether stands would serve as better rootstock sources for specific parts of the state. For each donor cell within the current species range, adaptedness values after hypothetical rootstock transfer were averaged across all recipient cells within existing walnut orchards within each respective section. These averages indicate how good the donor cell would be, on average, as a rootstock source for the Northern, Central, and Southern orchards, respectively.

Similarly, to identify existing croplands whose future climates rootstock from current wild walnut stands are likely preadapted to, the predicted adaptedness of potential rootstock sources if transferred to different future locations within existing cropland (Figure S8) was calculated for each species independently. For each climate scenario, adaptedness values were computed between every donor cell (raster grid cell within the current range of each species, representing a hypothetical rootstock source) and each recipient cell (raster grid cell within existing farmlands, representing a hypothetical planting location under future climate). The adaptedness values for each recipient cell (cells within existing farmland) after rootstock transfer were then averaged across all donor cells (cells within the current species range) to estimate how suitable that cropland would be, on average, as a planting site for each species.

## 3 Results

### 3.1 Genetic Structure and Variation

**FIGURE 2:**
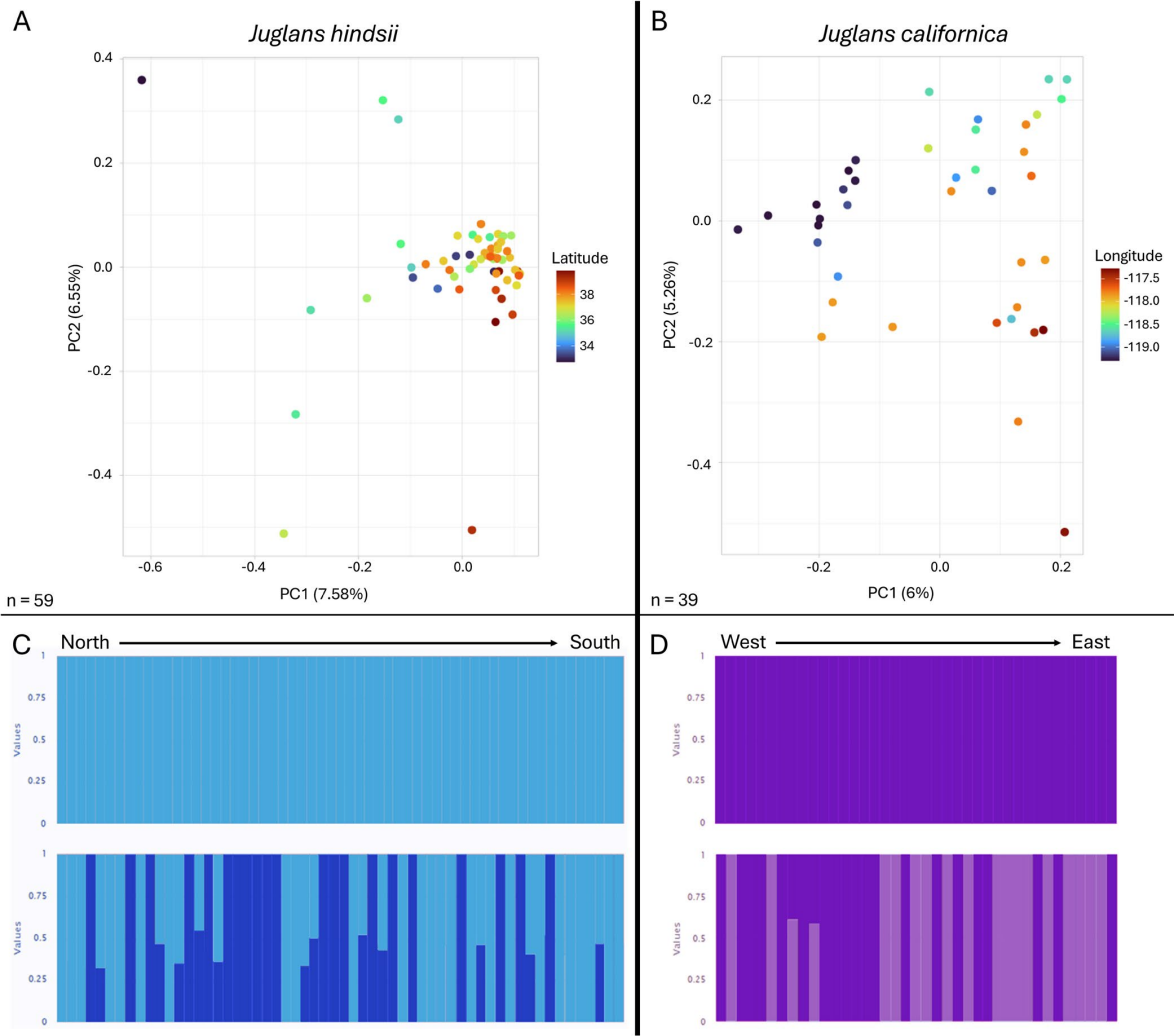
Analysis of genetic variation and structure of *J. hindsii* (A and C) and *J. californica* (B and D) populations based on whole genome sequences of adults shown in Figure 1. (A) PCA of *J. hindsii* (*n* = 59) with points coloured by latitude of collection site along the colour spectrum with the most northern site being dark red and most southern being dark blue, showing no clear patterns of genetic variation by location. (B) PCA of *J. californica* (*n* = 39) with points coloured by longitude of collection site along the colour spectrum with the most eastern site being dark red and most western being dark blue. A geographic gradient in genetic variation can be seen along PC2, with the axis spreading points east (bottom) to west (top), but might be explained with the slight signal of IBD in Figure S7. (C) ADMIXTURE plot of *J. hindsii* (*n* = 59) ordered north to south, testing for 1–10 genetic clusters (*K*), with K = 1–3 shown. *K* = 1 had the lowest CV error and thus was the most likely, with no discernable genetic gradients in higher Ks. (D) ADMIXTURE plot of *J. californica* (*n* = 39) ordered west to east, testing for 1–10 genetic clusters (*K*), with K = 1–3 shown. *K* = 1 had the lowest CV error and thus was the most likely, with no discernable genetic gradients in higher Ks.

The Principal Components Analysis (PCA) for *J. hindsii* (Northern California black walnut) did not reveal any discernable patterns of variation. PC axes 1 and 2 represented 7.58% and 6.55% of variation respectively, with individuals interspersed throughout (Figure 2A). ADMIXTURE gave the best clustering (*K*) of 1 based on CV error (Table S4), suggesting high gene flow (Sork and Smouse 2006) or recent dispersal. Forced higher values of K showed no patterns of genetic structure based on latitude (Figure 2C). We observed a minor trend in isolation by distance, with an *R* ^2^ value of 0.07 and *p* value of 0.001 (Figure S9A), indicating that genetic structure is not well explained by geographic distance. Similarly, we observed a nonsignificant trend in isolation by environment, with an R^2^ value of 0.001 and *p* value of 0.83 (Figure S9C), indicating that genetic structure is not well explained by environmental distance.

For *J. californica* (Southern California black walnut) PC axes 1 and 2, representing 6% and 5.26% of variation respectively, reveal a trend by longitude along PC2, with samples spreading west to east (Figure 2B). ADMIXTURE gave the best clustering (*K*) of 1 based on CV error (Table S5), again suggesting high gene flow or recent dispersal. Forced higher values of K show no patterns of genetic structure based on longitude (Figure 2D). We observed a negligible trend in isolation by distance, with an *R* ^2^ value of 0.02 and *p* value of 0.001 (Figure S9B), indicating that genetic structure is not well explained by geographic distance. Similarly, we observed a nonsignificant trend in isolation by environment, with an *R* ^2^ value of 0.001 and *p* value of 0.50 (Figure S9D), indicating that genetic structure is not well explained by environmental distance.

### 3.2 Climate‐Associated Loci and Patterns of Diversity

In *J. hindsii* , the multivariate, full redundancy analysis (RDA) including environment, geography, and population structure explained 28.6% of the variation in all loci with a *p* value of 0.003 and *R* ^2^ of 0.023 (Table S2). The constrained, partial RDA conditioned on geography and population structure found that environmental variables accounted for 68.4% of the explained variance with a *p* value of 0.096 and *R* ^2^ of 0.059 (Table S2), identifying 1405 climate‐associated SNPs as significant, the top 10% of which were kept for gene ontology and adaptedness analyses. Similarly high amounts of variance were explained by climate‐associated SNPs in other tree studies (DeSilva and Dodd 2020; Mead et al. 2024; Buck et al. 2026a, 2026b). When exploring the potential function associated with each of the 141 climate‐associated loci, 19 were located within ±1 kbp of a coding region, 12 of which had known functions, with five having associations to genes responsible for stress response, growth regulation, leaf development, and gene expression (Table S6).

In *J. californica* , the full RDA explained 39.1% of the variation in all loci with a *p* value of 0.001 and *R* ^2^ of 0.035 (Table S3). The constrained, partial RDA accounted for 67.5% of the explained variance with a *p* value of 0.131 and *R* ^2^ of 0.011 (Table S3), identifying 10,819 significant climate‐associated SNPs with the top 10% retained for gene ontology and adaptedness analyses. When exploring the potential function associated with each of the 1083 climate‐associated loci, 315 were located within ±1 kbp of a coding region, 191 had known functions, with 29 having associations to genes responsible for heat stress, defence, gene expression, and epigenetic regulation (Table S7).

**FIGURE 3:**
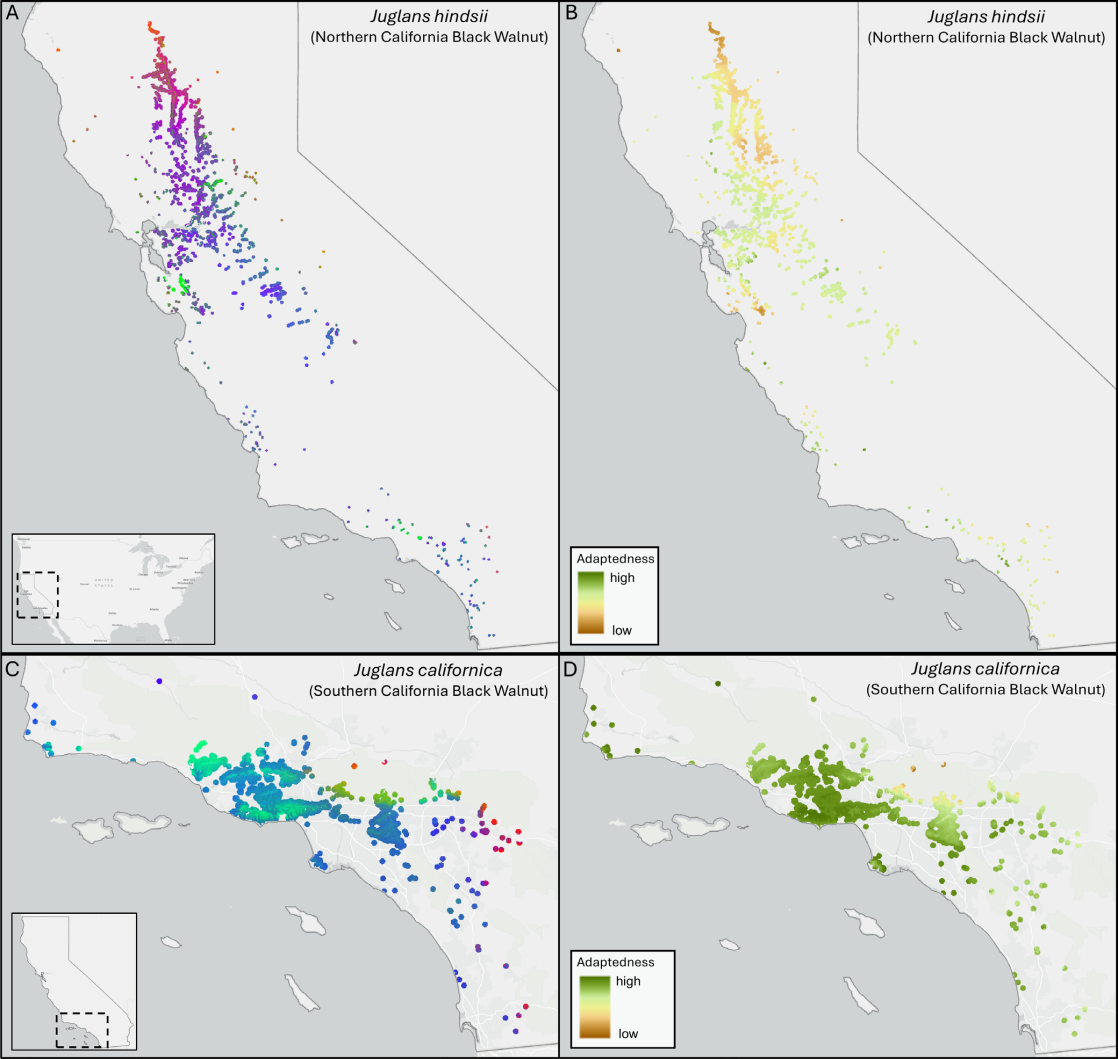
(A, C) Genomic turnover showing adaptive composition interpolated across the ranges of *J. hindsii* (A) and *J. californica* (C) by Gradient Forest models. Colours represent the genomic composition based on allele frequency turnover along environmental gradients. Similar colours (e.g., red and pink) indicate areas with similar adaptive composition, whereas greater differences in colours (e.g., red and blue) indicate larger differences in allele frequencies. (B, D) Adaptedness predictions of (B) *J. hindsii* 's and (D) *J. californica* 's ranges between recent and future climate models, averaging adaptedness values between the CNRM‐CM5 and HadGEM2‐ES scenarios for RCP‐8.5 in the 2070–2099 time period. Greener colours indicate higher climate adaptedness values, while browner colours indicate lower. (A‐D) Note that colours are relative to each species and not directly comparable between the two species because their GF models were trained with different datasets.

The Gradient Forest (GF) analyses based on climate‐associated allele frequencies identified by the RDA (Figures S5 and S6) revealed the spatial patterns of genomic turnover for each species (Figure 3A,C), which reflects the spatial distribution of putative local adaptation in each wild black walnut species. In Northern California black walnut, climate‐associated composition varied latitudinally across its range, with composition in the North being most different from the South (Figure 3A). In Southern California black walnut, climate‐associated composition varied across elevational gradients, with composition in higher elevation stands differing from lower elevation ones (Figure 3C). The importance of the different environmental variables in explaining putatively climate‐associated genetic variation was explored through Gradient Forest models independently for each species. For Northern California black walnut, soil pH had the highest importance, followed by percent clay, summer precipitation, soil depth, minimum temperature, winter precipitation, actual evapotranspiration, climate water deficit, available water capacity, and finally landform types (Figure S10A). For Southern California black walnut, summer precipitation had the highest importance, followed by landform types, winter precipitation, soil pH, percent clay, actual evapotranspiration, minimum temperature, climate water deficit, soil depth, and available water capacity (Figure S10B).

### 3.3 Adaptedness to Future Climate

For *J. hindsii* , the Gradient Forest models predicted a mosaic of adaptedness patterns, with stands more at risk of maladaptation to future climate in the northernmost extent of its range and south of San Francisco (Figure 3B). Stands with higher adaptedness are predicted in the western and southern extents of the Central Valley and along the coast of Southern California. Predictions varied by climate model, wherein the HadGEM2‐ES models predicted the lowest adaptedness in the northernmost extent of *J. hindsii* 's range and highest in the southernmost (Figure S11). The CNRM‐CM5 models predicted more heterogeneous patterns of adaptedness, with patches of low adaptedness in the northern extent of the Sacramento Valley and San Francisco Bay. For *J. californica* , the Gradient Forest models predicted relatively high adaptedness to future climate throughout its range, with stands in the San Gabriel and San Bernardino Mountains most at risk (Figure 3D). Patterns of adaptedness remained the same in both climate models, but the CNRM‐CM5 predicted lower levels of adaptedness overall (Figure S12).

### 3.4 Future Rootstock Sources for Existing Walnut Orchards

**FIGURE 4:**
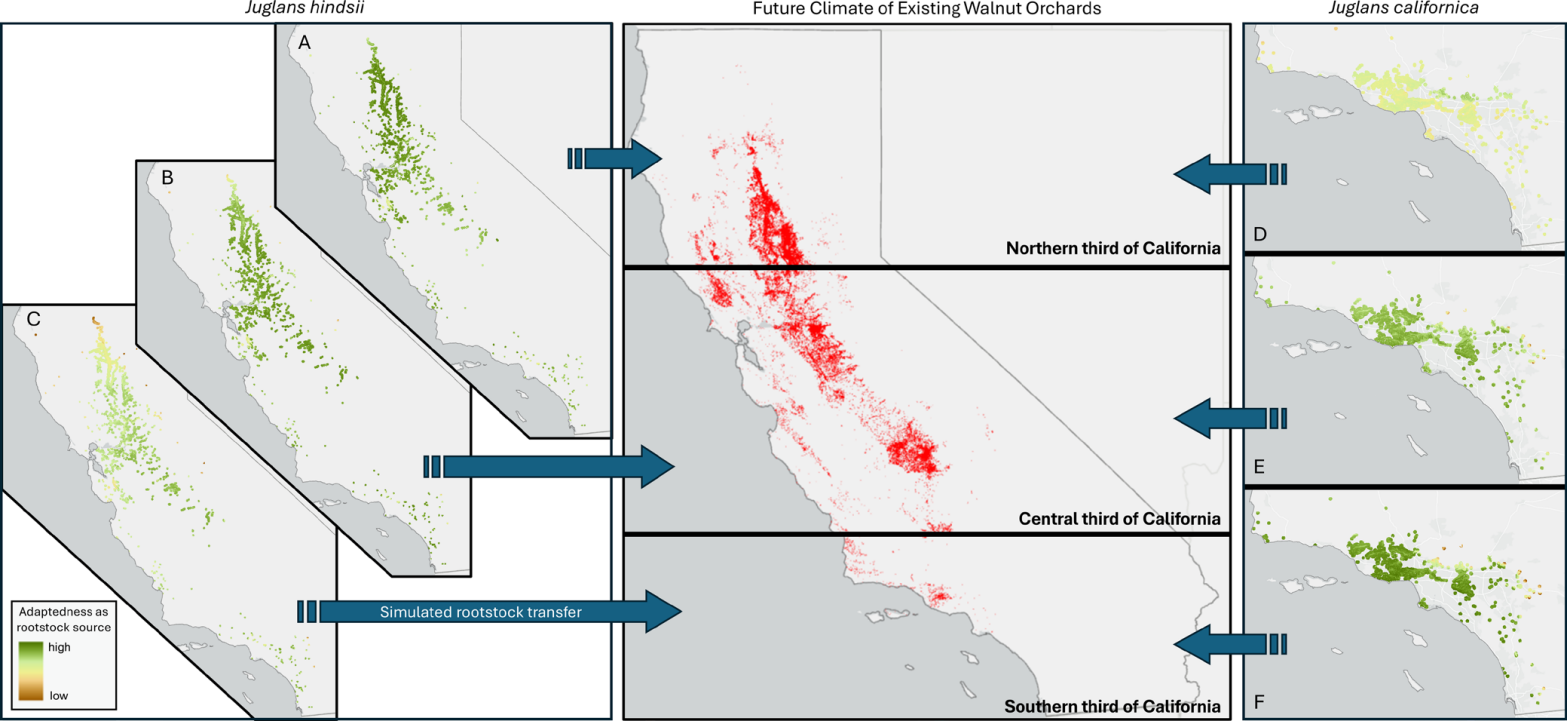
Average adaptedness of the walnut crop wild relatives, *J. hindsii* (A–C) and *J. californica* (D–F), if used as rootstock in the future climate of existing walnut orchards in the northern, central, and southern thirds of California (center panel). This metric gives farmers an idea of stands to test as potential rootstock sources in their orchards. Adaptedness values were averaged between the CNRM‐CM5 and HadGEM2‐ES scenarios for RCP‐8.5 in the 2070–2099 time period. Greener colours indicate higher climate adaptedness values, while browner colours indicate lower, but colours are not directly comparable between the two species because their GF models were trained with different datasets. Blue arrows represent simulated rootstock transfer from each black walnut species distribution (start) to existing walnut orchards (red) in each third of California.

When exploring potential rootstock sources for the future climate of existing walnut orchards, *J. hindsii* was predicted to have higher adaptedness if transferred to the northern and central thirds of California than for the southern third (Figure 4A–C). However, if transferred to walnut orchards in the southern third of California, stands in the southern extent of *J. hindsii* 's range were predicted to be more preadapted than the northern extent. *Juglans californica* was predicted to have opposite trends, with stands having highest adaptedness if transferred to the southern third of California walnut orchards than the central or northern thirds (Figure 4D–F). Interestingly, the stands within *J. californica* that were predicted to be worst if transferred to southern orchards have highest adaptedness if transferred to northern orchards. These overall trends can be compared between the two wild walnut species, although the adaptedness values are not directly comparable because their models were trained with different genomic and environmental data.

### 3.5 Future Growing Sites in Existing Cropland

**FIGURE 5:**
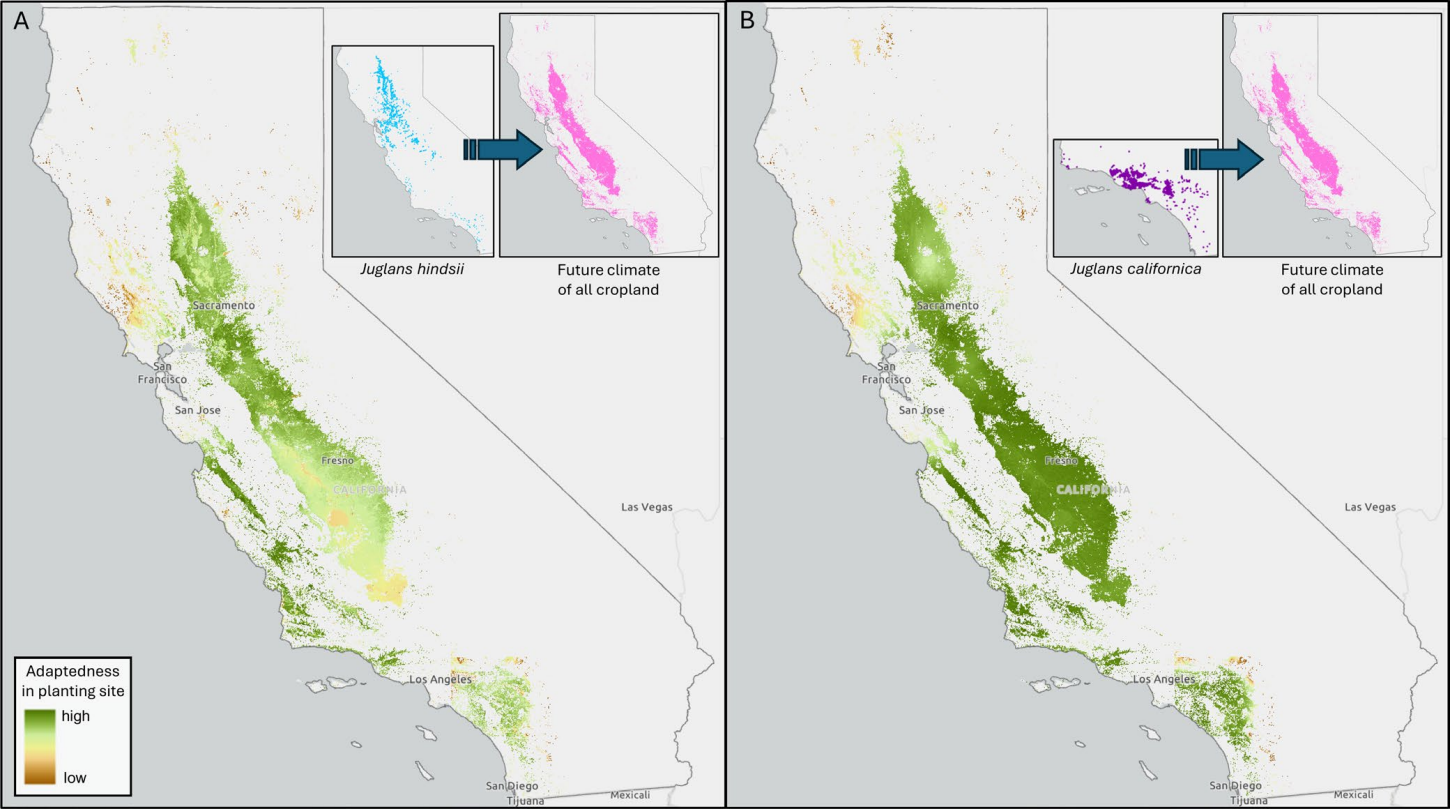
Average adaptedness of walnut crop wild relatives, *J. hindsii* (A) and *J. californica* (B), to future climate, if planted in all existing cropland in California. This metric gives farmers an idea of cropland that could potentially be converted to walnut orchards using each species as a rootstock. Adaptedness values were averaged between the CNRM‐CM5 and HadGEM2‐ES scenarios for RCP‐8.5 in the 2070–2099 time period. Greener colours indicate higher climate adaptedness values, while browner colours indicate lower, but colours are not comparable between the two species because their GF models were trained with different datasets. Blue arrows represent simulated rootstock transfer from each black walnut species distribution (start) to existing cropland (pink).

When identifying existing cropland that could be used as future planting sites for wild walnut rootstock, *J. hindsii* stands have the highest adaptedness to the future climate of croplands along the Central Coast and mid‐Central Valley directly south of Sacramento and the lowest adaptedness in patches in the lower‐Central Valley, mountains north of Los Angeles and in the northern Sierra and Coastal ranges (Figure 5A). *Juglans californica* stands have high adaptedness to the future climate of a majority of croplands and low adaptedness in small regions in the mountains north of Los Angeles and in the northern Sierra and Coastal ranges (Figure 5B). Overall, future planting sites for *J. hindsii* are more heterogeneous in adaptedness than for *J. californica* , but the species do share similar areas of low adaptedness in the cropland scattered far north and in the hills surrounding Los Angeles. Again, we note that overall trends can be compared between the two wild walnut species, but the scale of adaptedness values is not directly comparable due to differences in model inputs.

## 4 Discussion

Climate change is projected to reduce global crop yields, despite the need for a substantial increase in food production by 2050 to meet the demand of growing human population sizes (Tilman et al. 2011; United Nations 2017). New approaches are needed to identify and accelerate the development of climate‐resilient cultivars (Benitez‐Alfonso et al. 2023). Using two wild walnut species in California, we demonstrate that landscape genomic tools can help identify potential climate‐adapted rootstock. *Juglans hindsii* has the highest predicted adaptedness to the future climate of Northern and Central California walnut orchards, while *J. californica* has the highest predicted adaptedness to Southern and Central California walnut orchards. If walnut farmers wanted to test new rootstock sources for their existing orchards, they could start with this information to explore potential wild walnut stands. *Juglans californica* has high adaptedness to the future climate of more existing cropland than *J. hindsii* does, suggesting that *J. californica* may perform better in more cropland than *J. hindsii* . If farmers were looking to convert their farmland into walnut orchards with wild walnut rootstock, they could start with this information to explore potential sites. Below we discuss how landscape genomics can provide a tool for tree crop improvement.

### 4.1 Relative Risk of Wild Black Walnut Maladaptation to Future Climate

*Juglans hindsii* (Northern California black walnut) stands are more at risk of maladaptation to future climate in the northernmost extent of their range and south of San Francisco. Patterns of adaptedness varied by climate model used, with the warm/wet model, CNRM‐CM5, predicting two centres of low adaptedness mid‐range and south of San Francisco. The hot/dry climate model, HadGEM2‐ES, predicted more of a gradient of increasing adaptedness from north to south. The species exhibited high levels of local adaptation across its range, with climate‐associated composition being most different from north to south. Soil variables (pH and depth) were ranked as most important when describing climate‐associated genetic variation, but these two variables are not expected to change with climate. Their importance may potentially be related to *J. hindsii* 's riparian habitat, typically distributed along rivers and streams with soil pHs of 6–8 and a large preference for soil 200 cm deep. Perhaps the narrow range in environmental factors has put strong selective pressures on *J. hindsii* and has driven local adaptation. Moreover, its existence near perennial water sources may explain why precipitation variables do not rank higher despite the species' dependence on water.

*Juglans californica* (Southern California black walnut) stands in the San Gabriel mountains north of Los Angeles are more at risk of maladaptation to future climate than those in other parts of the species' range. Predictions seem to be more uniform both across its range and across climate models than predicted for *J. hindsii* , with slightly higher and more uniform adaptedness in the HadGEM2‐ES models than in the CNRM‐CM5s. The lower variation in adaptedness values predicted in *J. californica* could simply be due to the small size of its range, with less environmental variation and less predicted climate change, but the latter would need to be examined to confirm this. While a small area of *J. californica* 's range is predicted to be most at risk of maladaptation to future climate, any further reduction in population sizes could imperil the already Near Threatened species. Despite its small range, *J. californica* exhibited high levels of local adaptation, especially along elevational gradients and among the edges of its known range. Notably, *J. californica* occupies habitat with higher climate water deficits (less water availability) than *J. hindsii* , suggesting that this species has adapted to the drier climates of Southern California and the presence of ephemeral streams. Its habitat, higher adaptedness in the hotter/drier climate model, and summer precipitation explaining the most climate‐associated variation potential indicate *J. californica* may be more adapted to hotter and drier climates than *J. hindsii* .

### 4.2 Identifying Potential Wild Rootstock Sources for Existing Walnut Orchards

Due to the increasing effects of climate change and water scarcity, new approaches are needed to identify andaccelerate the breeding of climate‐resilient cultivars (Benitez‐Alfonso et al. 2023). Crop wild relatives can introduce genetic diversity and adaptive traits to cultivars (Hajjar and Hodgkin 2007; Choudhary et al. 2017; Kapazoglou et al. 2023), especially when used as rootstock, where they can provide instant physiological benefits without genetic changes (Koepke and Dhingra 2013). With the untested future climate resilience of current walnut rootstock, we explored the potential of replacing rootstock in existing walnut orchards with wild *J. hindsii* and *J. californica* . Unsurprisingly, wild *J. hindsii* stands are better rootstock sources for the northern and central third of California than for the southern third. If walnut breeders are exploring the use of *J. hindsii* as a rootstock source, they could consider choosing the origin of the rootstock based on the location of the intended orchard. The geographic origin of the *J. hindsii* parent used to create one of the most commonly cloned Paradox hybrid rootstocks, Vlach, is Modesto (Central California), while the origin for the clonal VX211 rootstock was not reported (Buzo et al. 2009), yet both are widely used in walnut orchards across California. Orchards in Central California may have higher adaptedness using Vlach Paradox rootstock, but farmers in Northern and Southern California may want to explore using Paradox rootstock created with northern and southern *J. hindsii* stands, respectively.

In contrast, wild *J. californica* stands may be better rootstock sources for the southern third of California than the central and more notably, the northern third. The expected direction of climate change, and consequently distribution shifts, is generally polewards (Parmesan and Yohe 2003; Franke et al. 2022), so it is unexpected that *J. californica* stands are not predicted to have higher adaptedness in more northern walnut orchards. This observed pattern could be due to local adaptations to current Southern California climates that are not similar to the predicted future of more northern walnut orchards, but analyses comparing the two climates would need to be done to support this hypothesis. If walnut breeders are exploring the use of *J. californica* as a rootstock source, they too should consider the origin of the rootstock based on the location of their orchards. *Juglans californica* has not been tested as a rootstock or as a hybrid parent with *J. regia* , so its effects on nut production and disease resistance are unknown, but it may be worthwhile to at least explore its potential as climate adapted rootstock. Crosses between *J. regia* and *J. californica* should be considered, as they are expected to hybridize readily given the ease of hybridization that *J. hindsii* has with *J. regia* to create the Paradox rootstock, *J. hindsii* and *J. californica* 's close phylogenetic relationship (Fjellstrom and Parfitt 1995; Stanford et al. 2000; Ebrahimi et al. 2025), and the recorded hybridization between *J. hindsii* and *J. californica* (Zapata 2024).

Due to the different genomic and environmental data used to train each species' Gradient Forest models, the adaptedness values are not comparable between species, so we cannot say which species would have higher adaptedness in specific orchards than the other. Further, because the adaptedness predictions are relative and unitless, the exact fitness consequences of predicted adaptedness values are unknown, and we thus cannot say how beneficial specific stands could be as rootstock sources. Several studies have found a correlation between adaptedness and fitness (Fitzpatrick et al. 2021; Láruson et al. 2022; Gain et al. 2023; Lind and Lotterhos 2024; Archambeau et al. 2026; Verrico et al. 2026), suggesting that the use of genomic tools may be a good first step to improving crop resilience. However, too few studies are currently available to determine if these correlations are universal, and only one study to date has explored this in a crop species (Gain et al. 2023). So, until the approach is more fully validated, breeders should test potential rootstocks from a variety of geographical sources to determine if transferred genotypes will increase fitness. However, we demonstrate here that if breeders are looking for alternative rootstock sources within wild walnut relatives, landscape genomic tools can identify which stands of each species would have higher adaptedness in the future climate of existing walnut orchards.

We also acknowledge the full replacement of orchards with new rootstock is drastic and intensive, especially given the time commitment required to establish new trees and the commercial value lost while waiting for seedlings to mature to fruiting age. However, when trees senesce and need replacement, the investment in new rootstock for future climate could be profitable. Moreover, as young trees are establishing, the temporary loss of some production may outweigh the declining yield and even crop failure due to climate change (Vuppalapati 2023). Irrigation could prevent some decline in yield (Lobell et al. 2006), but California's recurrent water scarcity issues (Diffenbaugh et al. 2015) and the already high water use by the agriculture sector (Mount and Hanak 2019) do not make increased irrigation a reliable solution. Now might be a good time to look for walnut genotypes that can tolerate less water. Given the preliminary and general nature of our analyses, the consequences of using specific stands as rootstock sources should be tested before widespread implementation in orchards. Combining the selection of heat resistant *J. regia* accessions (as exemplified in Momayyezi et al. 2025) and drought tolerant rootstock may improve results even further.

### 4.3 Identifying Potential Cropland for Planting Wild Walnut Rootstock

If farmers were interested in converting their existing cropland into orchards with wild black walnut rootstock, our results demonstrate how using landscape genomic tools can identify croplands that would be better suited for each species of wild walnut. *Juglans hindsii* stands along the Central Coast and mid‐Central Valley directly south of Sacramento appear most preadapted to the future climate of croplands, whereas *J. californica* stands are most preadapted to future climates of a majority of croplands along the Central Coast, within the Central Valley, and even near Los Angeles. Both species share high adaptedness to the future climate of cropland along the Central Coast and low adaptedness to the scattered cropland in the far north and along the periphery of Southern California. In cases of low adaptedness, farmers may want to avoid crop conversion or find other crop species that are more suitable. Alternatively, farmers may want to explore alternative planting sites outside of current existing cropland by examining future species distribution models (also referred to as ecological niche models). Such models are available for *J. californica* and show high habitat suitability in higher elevation montane regions (Rose et al. 2023). These species distribution models could identify potentially suitable future planting sites for either species, but we caution that they do not take local adaptation into account so recommend that these explorations also include genome‐informed methods (Buck et al. 2026b).

These results should not be taken as endorsing the conversion of existing cropland to walnut orchards. Walnuts take five to eight years to mature, with full crop production at 12–16 (Thompson 1961). They can live up to 200 years (Reeve 2022), with some orchards having trees almost 100 years old (Vuppalapati 2023). This long‐term investment may make walnut growers less likely to relocate to more suitable growing locations. We also recognize the water use requirements of walnut crops are high (Goldhamer et al. 1988; Vanham et al. 2020). Although we are demonstrating first steps to find climate adapted wild walnuts, they may still require more water than other crops already growing in fields (Mekonnen and Hoekstra 2011; Vanham et al. 2020). Farmers may want to explore additional methods to reduce water use, such as regulated irrigation deficit, in which provided water is reduced during less sensitive growth phases (Calvo et al. 2023). Yet, neither the Paradox nor *J. hindsii* rootstocks responded well to reduced irrigation, producing significantly fewer nuts than fully watered trees (Buchner et al. 2008). Because our models did not incorporate irrigation as a variable, it is possible that the wild walnuts could require less irrigation, or that differences in adaptedness predictions could become more substantial or inconsequential with supplemented water. Thus, we emphasize tests are needed to validate the impact of irrigation.

### 4.4 Additional Caveats to Our Study

In addition to limitations described in previous sections, this study has further caveats. First, landscape genomic analyses require large sample sizes to capture environmental and genetic gradients (Sork et al. 2013; Bishop et al. 2025). We acknowledge the sampling for both species was low, although less severe for *J. californica*, which has a more restricted distribution, of which our sampling covered a majority. Nonetheless, sample size can explain the low statistical support for any partial redundancy analyses. It is also possible the low support for environmental variables explaining genomic variation could be due to a lack of strong selection gradients across *J. californica* 's limited range. In both cases, more sampling could increase statistical power, possibly leading to the detection of stronger patterns of local adaptation.

A second caveat concerns the nature of rootstock biology. The adaptedness values for rootstock transfer are generated through genome‐environment associations that assume local adaptation as a result of selection on an entire tree, not just the rootstock. It is likely that selection on both above and below ground traits have shaped patterns of local adaptation that consequently influence our predictions. The adaptedness predictions here are thus modelled for an entire tree, yet only the rootstock will be used in orchards. This complexity might affect modelling genetic chimeras, wherein the composition of two different genomes may interact and function as a single organism. Not only can the inherent traits of the rootstock (i.e., water acquisition or disease resistance) affect the scion's fitness (i.e., growth and nut production), but the rootstock can also express traits in the scion itself (e.g., dwarfing; Koepke and Dhingra 2013). As a result, the outcomes of adaptedness may differ when the rootstock is combined with another species' scion. Further, hybrid rootstocks (e.g., Paradox, the newly proposed *J. regia* × *J. californica* , or even *J. hindsii* × *J californica*) were not modelled, but the untested combinations of genotypes could create novel rootstocks with introgressed adaptive traits (Kapazoglou et al. 2023). Because hybrid rootstock will be tested before use, the extent of climate adaptations and disease resistance can be assessed.

A third caveat concerns the mapping approach of our analysis. To help visualize the thin range of both wild walnut species, we added a 2.5 km buffer around existing occurrence data. Therefore, the adaptedness predictions are displaying slightly larger areas than either wild walnut occupies. However, models were trained on climatic and genomic data from existing trees, so it is expected that the predictions are useful where walnut does exist.

A fourth caveat concerns applications to novel environments. Adaptedness predictions can become less accurate when extrapolating to environments outside of the range of climate data used in model training (Lind and Lotterhos 2024). Here, rootstock transfer was modelled into areas outside of both wild walnuts' existing ranges, especially for *J. californica* , so the effects of rootstock transfer should be interpreted with caution until empirical studies are done.

A final caveat concerns the generality of our predictions. For heuristic value, we model the average adaptedness of an entire species in the future of planting sites across existing walnut orchards. However, the size of both the rootstock source regions and planting sites can be scaled up or down to match a farmer's needs and can be implemented on a single grid cell to range‐wide scales. Doing so without an identified rootstock source or planting site would result in a near‐infinite number of possible combinations. From a practical point of view, a farmer is more likely to focus on the land they own, in which case it would be more prudent to identify rootstock sources that match the future climate of that specific planting site rather than the entirety of California.

## 5 Conclusions

Climate change is expected to lower global crop yields, even as food production must increase substantially to meet growing demand, underscoring the need for climate‐resilient cultivars. Using two wild crop relative walnut species in California, we demonstrate that landscape genomic tools can identify climate‐adapted rootstock for walnut production. Our results suggest that *J. hindsii* will be best suited for future climates of Northern and Central California walnut orchards, while *J. californica* is best suited for Southern and Central California orchards. *Juglans californica* is predicted to match future climates across more existing cropland than *J. hindsii* does, suggesting that *J. californica* may perform better in more cropland than *J. hindsii* . These insights offer an initial guide for growers and breeders exploring wild rootstocks. In addition to their economic value, wild California walnuts support riparian ecosystems and deserve protection for their ecological and cultural value. Overall, this study highlights the added importance of conserving natural populations and shows how landscape genomics can identify climate‐ready genotypes for future testing and deployment.

## Author Contributions

R.C.B.: designed research, performed research, analysed data, wrote and edited paper; D.J.Z.: designed research, collected samples, performed research; V.L.S.: designed research, acquired funding, provided resources, wrote and edited paper.

## Funding

This work was supported by the California Conservation Genomics Project with funding provided to the University of California by the State of California, State Budget Act of 2019 [UC Award ID RSI‐19‐690224]; The Nature Conservancy, and the La Kretz Center for California Conservation Science.

## Disclosure

Benefit‐Sharing Statement: This study did not involve genetic resources subject to international access and benefit‐sharing obligations under the Nagoya Protocol.

## Conflicts of Interest

The authors declare no conflicts of interest.

## Supporting information


**Table S1:** Ten environmental variables used to associate genomic variation with environment, sourced at 270 m from the Basin Characterization model (Flint et al. 2021; Stern et al. 2024), gNATSGO (Soil Survey Staff 2020), and Theobald et al. (2015), all provided by Rose et al. (2023).
**Table S2:** Partial redundancy analysis of all variants found in the whole genome sequences of 53 Northern California black walnut adults sampled throughout the species range, testing the effects of environment, geography, population structure and all three on genetic variation (W = genomic data; env = ten environmental variables; geo = latitude, longitude and latitude × longitude; pop = neutral population structure represented by PC1).
**Table S3:** Partial redundancy analysis of all variants found in the whole genome sequences of 39 Southern California black walnut adults sampled throughout the species range, testing the effects of environment, geography, population structure and all three on genetic variation (W = genomic data; env = ten environmental variables; geo = latitude, longitude, and latitude × longitude; pop = neutral population structure represented by PC1).
**Table S4:** CV values from ADMIXTURE for *J. hindsii* , with the lowest CV error indicating the best K number of clusters. Our analyses show *K* = 1 (bold) as the best *K*.
**Table S5:** CV values from ADMIXTURE for *J. californica* , with the lowest CV error indicating the best K number of clusters. Our analyses show *K* = 1 (bold) as the best *K*.
**Figure S1:** Ranges of Northern California black walnut ( *J. hindsii* ; blue) and Southern California black walnut ( *J. californica* ; purple) with all sampled individuals (points) coloured by species identity from the ADMIXTURE results in Figure S3. The overlaid map is an enlarged portion of the two species' range overlap.
**Figure S2:** Environmental gradients of Northern California black walnut captured by our sampling design (red) vs. the entire species distribution of *J. hindsii* (blue) in California, USA. Values of each environmental variable are on the *x*‐axis, while density of those values are on the *y*‐axis.
**Figure S3:** Environmental gradients of Southern California black walnut captured by our sampling design (red) vs. the entire species distribution of *J. californica* (blue) in California, USA. Values of each environmental variable are on the *x*‐axis, while density of those values are on the *y*‐axis.
**Figure S4:** Preliminary ADMIXTURE plot of all *Juglans* samples, including Northern and Southern California black walnuts, used to genetically identify samples before genotype‐environment analyses. *K* = 2 had the lowest CV error and thus was the most likely, with admixed individuals removed for downstream analyses.
**Figure S5:** Distribution of *J. hindsii* genomic variation in RDA space using all loci, with RDA1 accounting for 13.53% of variation and RDA2 accounting for 12.33%. The 1405 outlier loci above the Bonferroni‐corrected significance threshold (α = 0.01/n) are coloured orange, with the highest 10% of those *p* values (141 loci) coloured purple, while the other 99,123 loci are coloured grey. The strength of each environmental variable on variation is represented by the blue loading arrows, with longer arrows representing more importance in explaining variation in the direction they are pointing.
**Figure S6:** Distribution of *J. californica* genomic variation in RDA space using all loci, with RDA1 accounting for 11.2% of variation and RDA2 accounting for 10.66%. The 10,819 outlier loci above the Bonferroni‐corrected significance threshold (α = 0.01/*n*) are coloured orange, with the highest 10% of those *p* values (1083 loci) coloured purple, while the other 704,456 loci are coloured grey. The strength of each environmental variable on variation is represented by the blue loading arrows, with longer arrows representing more importance in explaining variation in the direction they are pointing.
**Figure S7:** Map of existing walnut orchards (brown) in California. Extracted from the USDA Cropland Data Layer.
**Figure S8:** Map of all existing cropland types (pink) in California. Extracted from the USDA Cropland Data Layer.
**Figure S9:** (A and B) Isolation by distance results showing geographic distance in kilometres and genetic distance (as the Euclidean distance between individual genotypes) with each dot representing a pairwise comparison between individuals. (A) For Northern California black walnut ( *J. hindsii* ), genetic distance has a slight trend (R^2^ = 0.073) with geographic distance, showing minor evidence of isolation by distance (*p* = 0.001). (B) For Southern California black walnut ( *J. californica* ), genetic distance has a slight trend (R^2^ = 0.021) with geographic distance, showing minor evidence of isolation by distance (*p* = 0.001). (C and D) Isolation by environment accounting for geography showing environmental distance (calculated as the Euclidean distances of scaled mean environmental variables at each individual's location) and genetic distance (calculated as the Euclidean distance between individual genotypes) with each dot representing a pairwise comparison between individuals. (C) For Northern California black walnut, genetic distance has a nonsignificant trend (*R* ^2^ = 0.001, *p* = 0.81) with environmental distance, showing no evidence of isolation by environment. (D) For Southern California black walnut, genetic distance has a nonsignificant trend (*R* ^2^ = 0.001, *p* = 0.5) with environmental distance, showing no evidence of isolation by environment.
**Figure S10:** Importance of environmental variables in the gradient forest models for wild Northern (A) and Southern (B) California black walnuts. Each graph shows the split importance weighted by the variance explained for each locus. Soil pH is the most important variable in explaining putative adaptive variation in Northern California black walnut, while summer precipitation is the most important for Southern California black walnut.
**Figure S11:** Climate adaptedness predictions for *J. hindsii* using Gradient Forest for climate models HadGEM2‐ES and CNRM‐CM5, RCP 4.5 and 8.5, and 30‐year average time periods 2040–2069 and 2070–2099. Greener colours indicate higher climate adaptedness values.
**Figure S12:** Climate adaptedness predictions for *J. californica* using Gradient Forest for climate models HadGEM2‐ES and CNRM‐CM5, RCP 4.5 and 8.5, and 30‐year average time periods 2040–2069 and 2070–2099. Greener colours indicate higher climate adaptedness values.

**Table S6:** mec70420‐sup‐0002‐TableS6.xlsx. *J. hindsii* gene ontology of climate‐associated SNPs within 1 kbp of a known gene.

**Table S7:** mec70420‐sup‐0003‐TableS7.xlsx. *J. californica* gene ontology of climate‐associated SNPs within 1kbp of a known gene.

## Data Availability

Raw sequencing reads and metadata are available at NCBI under BioProjects PRJNA1437785 and PRJNA1437786 (https://www.ncbi.nlm.nih.gov/bioproject/1437785 and https://www.ncbi.nlm.nih.gov/bioproject/1437786). Locality data for collected samples are available in the metadata for sequencing reads. Analysis scripts are available at Github (https://github.com/ryancollinbuck/juglans) and archived at Dryad (https://datadryad.org/dataset/10.5061/dryad.jwstqjqqx).
