# Supplementary material for: Landscape Genomic Tools Can Inform Future Rootstock and Farmland Selection for an Agricultural Tree Nut From Its Wild Relatives
Source: Mol Ecol. 2026 Jun 12;35(11):e70420. doi: 10.1111/mec.70420 (PMC13261541; doi:10.1111/mec.70420)
Supplement: Supplementary file 1 — Table S1: Ten environmental variables used to associate genomic variation with environment, sourced at 270 m from the Basin Characterization model (Flint et al. 2021; Stern et al. 2024), gNATSGO (Soil Survey Staff 2020), and Theobald et al. (2015), all provided by Rose et al. (2023). Table S2: Partial redundancy analysis of all variants found in the whole genome sequences of 53 Northern California black walnut adults sampled throughout the species range, testing the effects of environment, geography, population structure and all three on genetic variation (W = genomic data; env = ten environmental variables; geo = latitude, longitude and latitude × longitude; pop = neutral population structure represented by PC1). Table S3: Partial redundancy analysis of all variants found in the whole genome sequences of 39 Southern California black walnut adults sampled throughout the species range, testing the effects of environment, geography, population structure and all three on genetic variation (W = genomic data; env = ten environmental variables; geo = latitude, longitude, and latitude × longitude; pop = neutral population structure represented by PC1). Table S4: CV values from ADMIXTURE for J. hindsii , with the lowest CV error indicating the best K number of clusters. Our analyses show K = 1 (bold) as the best K. Table S5: CV values from ADMIXTURE for J. californica , with the lowest CV error indicating the best K number of clusters. Our analyses show K = 1 (bold) as the best K. Figure S1: Ranges of Northern California black walnut ( J. hindsii ; blue) and Southern California black walnut ( J. californica ; purple) with all sampled individuals (points) coloured by species identity from the ADMIXTURE results in Figure S3. The overlaid map is an enlarged portion of the two species' range overlap. Figure S2: Environmental gradients of Northern California black walnut captured by our sampling design (red) vs. the entire species distribution of J. hindsii (blue) in California, [file MEC-35-e70420-s001.docx]

**SUPPLEMENTARY INFORMATION FOR:**

Landscape genomic tools can inform future rootstock and farmland selection for an agricultural tree nut from its wild relatives

Ryan C. Buck^1,2,^*, Diego J. Zapata^1^, and Victoria L. Sork^1,3,^*

**Table S1**. Ten environmental variables used to associate genomic variation with environment, sourced at 270m from the Basin Characterization model (Flint et al., 2021; Stern et al., 2024), gNATSGO (Soil Survey Staff, 2020), and Theobald et al. (2015), all provided by Rose et al. (2023).

| **Variable abbreviation** | **Variable full name** | **Units** | **Variable type** | **Description** |
| --- | --- | --- | --- | --- |
| AET | Actual evapotranspiration | mm | Hydrologic | Amount of water that evaporates from the surface and is transpired by plants, summed annually |
| AWC | Available water capacity | mm | Soil | The amount of water that an increment of soil depth, inclusive of fragments, can store that is available to plants |
| CWD | Climate water deficit | mm | Hydrologic | Annual evaporative demand that exceeds available water, summed annually |
| Depth | Soil depth | cm | Soil | Soil depth to bedrock |
| PCT_clay | Percent clay | % | Soil | Mineral particles less than 0.002mm in equivalent diameter as a weight percentage of the less than 2.0mm fraction |
| PH | Soil pH | pH | Soil | Measure of soil acidity or alkalinity |
| PPT_jja | Summer precipitation | mm | Climate | Total monthly summer precipitation (rain or snow), summed annually across June, July, and August |
| PPT_djf | Winter precipitation | mm | Climate | Total monthly winter precipitation (rain or snow), summed annually across December, January, and February |
| Terrain | Landform types | N/A | Terrain | 15 categorical hillslope positions defined by their topographic position index, slope, and continuous heat index |
| TMN | Minimum temperature | °C | Climate | Minimum monthly temperature, averaged annually |

**Table S2**. Partial redundancy analysis of all variants found in the whole genome sequences of 53 Northern California black walnut adults sampled throughout the species range, testing the effects of environment, geography, population structure, and all three on genetic variation (W = genomic data; env = ten environmental variables; geo = latitude, longitude, and latitude x longitude; pop = neutral population structure represented by PC1).

| **pRDA model** | **R^2^** | **Inertia** | **P(>F)** | **Proportion of explainable variance** | **Proportion of total variance** |
| --- | --- | --- | --- | --- | --- |
| Full : W ~ env + geo + pop | 0.0225 | 4366 | 0.003 | 1 | 0.286 |
| Pure Climate : W ~ env \| (geo + pop) | 0.0587 | 2988 | 0.096 | 0.684 | 0.196 |
| Pure Geography : W ~ geo \| (env + pop) | 0.0030 | 897.7 | 0.296 | 0.206 | 0.059 |
| Pure Structure : W ~ pop \| (env + geo) | 0.0115 | 419.1 | 0.002 | 0.096 | 0.027 |
| Confounded (env / geo / pop) |  | 61.2 |  | 0.014 | 0.004 |
| Total unexplained |  | 10920 |  |  | 0.714 |
| Total inertia |  | 15280 |  |  | 1 |

**Table S3**. Partial redundancy analysis of all variants found in the whole genome sequences of 39 Southern California black walnut adults sampled throughout the species range, testing the effects of environment, geography, population structure, and all three on genetic variation (W = genomic data; env = ten environmental variables; geo = latitude, longitude, and latitude x longitude; pop = neutral population structure represented by PC1).

| **pRDA model** | **R^2^** | **Inertia** | **P(>F)** | **Proportion of explainable variance** | **Proportion of total variance** |
| --- | --- | --- | --- | --- | --- |
| Full : W ~ env + geo + pop | 0.0350 | 39570 | 0.001 | 1 | 0.391 |
| Pure Climate : W ~ env \| (geo + pop) | 0.0108 | 26700 | 0.131 | 0.675 | 0.264 |
| Pure Geography : W ~ geo \| (env + pop) | 0.0074 | 8250 | 0.240 | 0.208 | 0.081 |
| Pure Structure : W ~ pop \| (env + geo) | 0.0069 | 3033 | 0.169 | 0.077 | 0.030 |
| Confounded (env / geo / pop) |  | 1587 |  | 0.040 | 0.016 |
| Total unexplained |  | 61750 |  |  | 0.610 |
| Total inertia |  | 101300 |  |  | 1 |

**Table S4.** CV values from ADMIXTURE for *J. hindsii*, with the lowest CV error indicating the best K number of clusters. Our analyses show K=1 (bold) as the best K.

| K | CV Error |
| --- | --- |
| **1** | **0.35999** |
| 2 | 0.41213 |
| 3 | 0.43622 |
| 4 | 0.48670 |
| 5 | 0.53627 |
| 6 | 0.55938 |
| 7 | 0.57452 |
| 8 | 0.61554 |
| 9 | 0.66788 |
| 10 | 0.73007 |

**Table S5.** CV values from ADMIXTURE for *J. californica*, with the lowest CV error indicating the best K number of clusters. Our analyses show K=1 (bold) as the best K.

| K | CV Error |
| --- | --- |
| **1** | **0.38422** |
| 2 | 0.44001 |
| 3 | 0.48598 |
| 4 | 0.53269 |
| 5 | 0.57506 |
| 6 | 0.62254 |
| 7 | 0.69333 |
| 8 | 0.75838 |
| 9 | 0.83080 |
| 10 | 0.90204 |


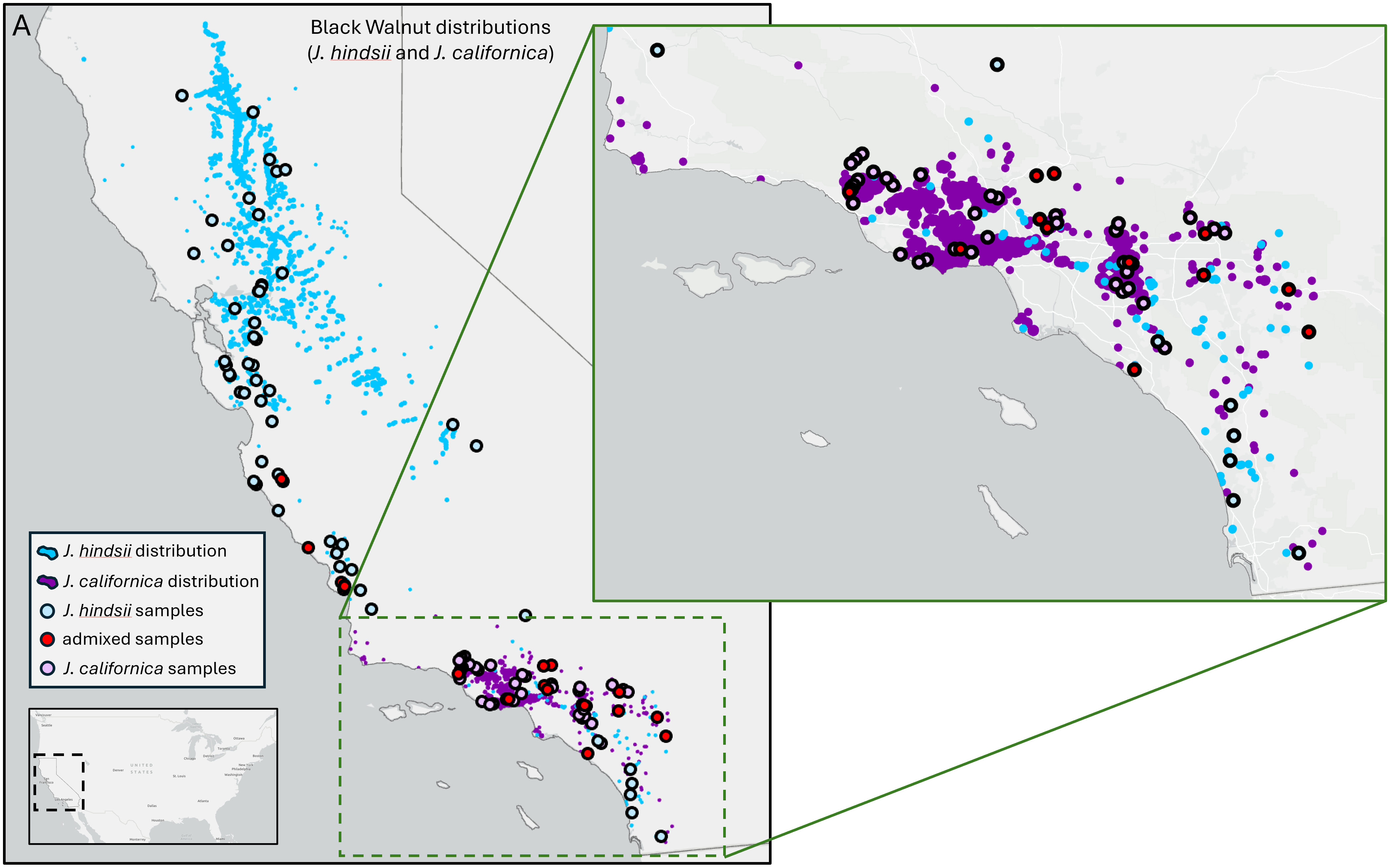


**Figure S1.** Ranges of Northern California black walnut (*J. hindsii*; blue) and Southern California black walnut (*J. californica*; purple) with all sampled individuals (points) colored by species identity from the ADMIXTURE results in Figure S3. The overlaid map is an enlarged portion of the two species’ range overlap.


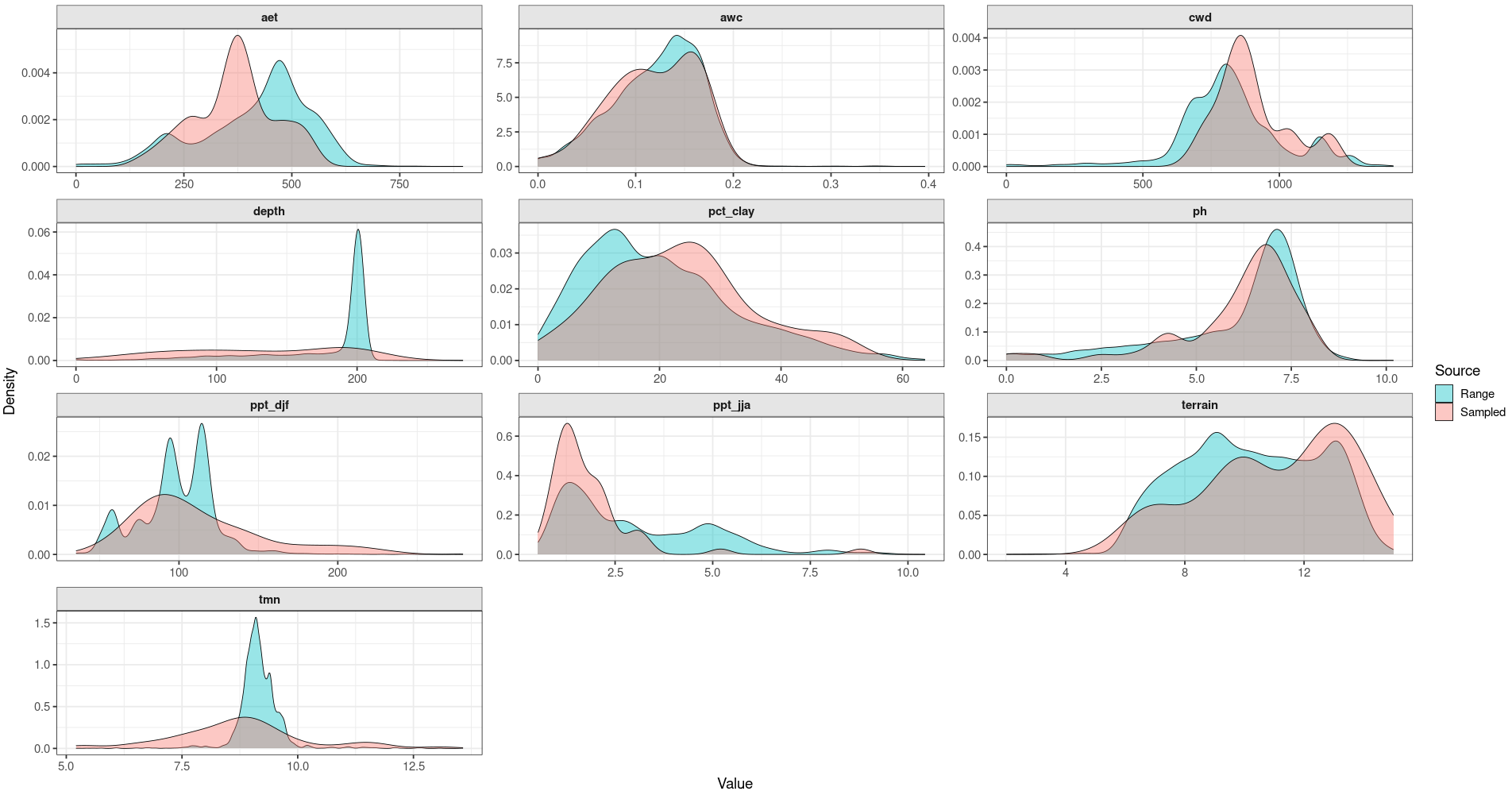
 **Figure S2.** Environmental gradients of Northern California black walnut captured by our sampling design (red) vs the entire species distribution of *J. hindsii* (blue) in California, USA. Values of each environmental variable are on the x-axis, while density of those values are on the y-axis.


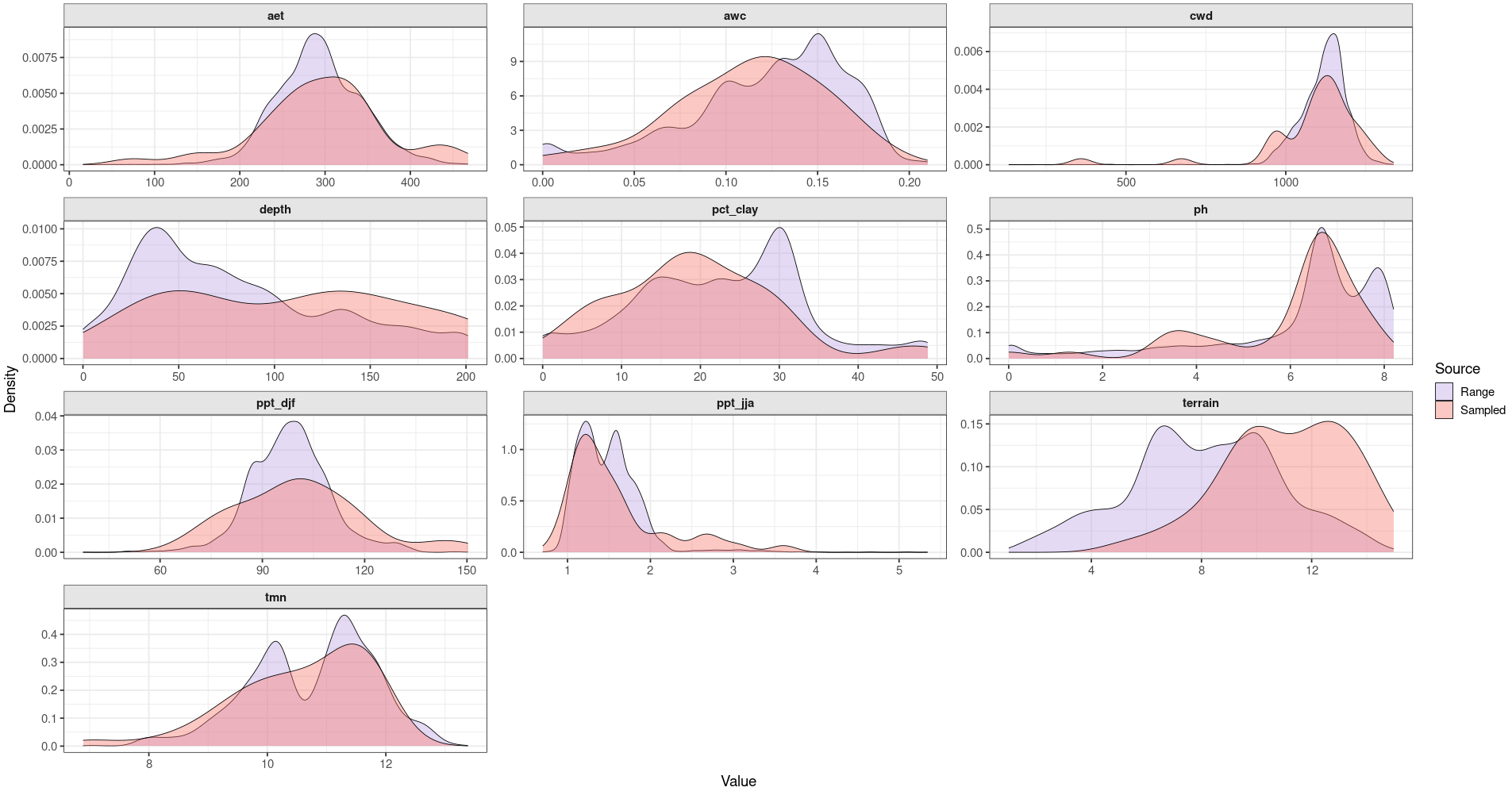
 **Figure S3.** Environmental gradients of Southern California black walnut captured by our sampling design (red) vs the entire species distribution of *J. californica* (blue) in California, USA. Values of each environmental variable are on the x-axis, while density of those values are on the y-axis.

**
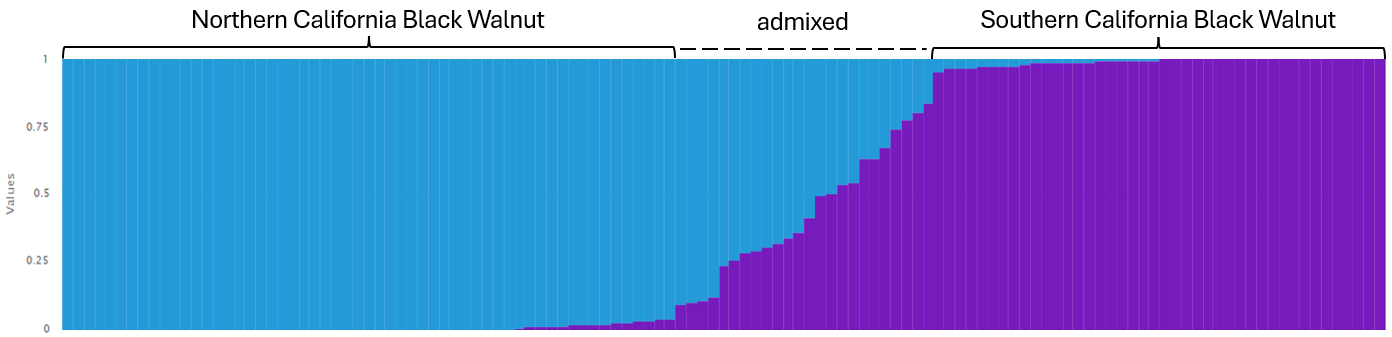
Figure S4.** Preliminary ADMIXTURE plot of all *Juglans* samples, including Northern and Southern California black walnuts, used to genetically identify samples before genotype-environment analyses. K=2 had the lowest CV error and thus was the most likely, with admixed individuals removed for downstream analyses.

**
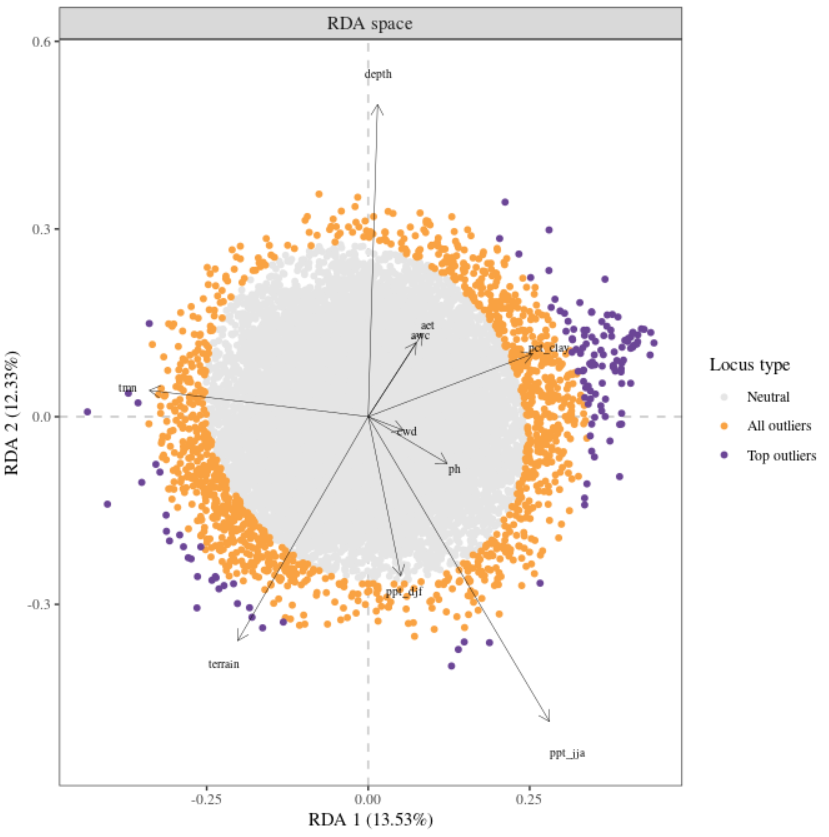
 Figure S5.** Distribution of *J. hindsii* genomic variation in RDA space using all loci, with RDA1 accounting for 13.53% of variation and RDA2 accounting for 12.33%. The 1,405 outlier loci above the Bonferroni-corrected significance threshold (α=0.01/n) are colored orange, with the highest 10% of those p-values (141 loci) colored purple, while the other 99,123 loci are colored grey. The strength of each environmental variable on variation is represented by the blue loading arrows, with longer arrows representing more importance in explaining variation in the direction they are pointing.
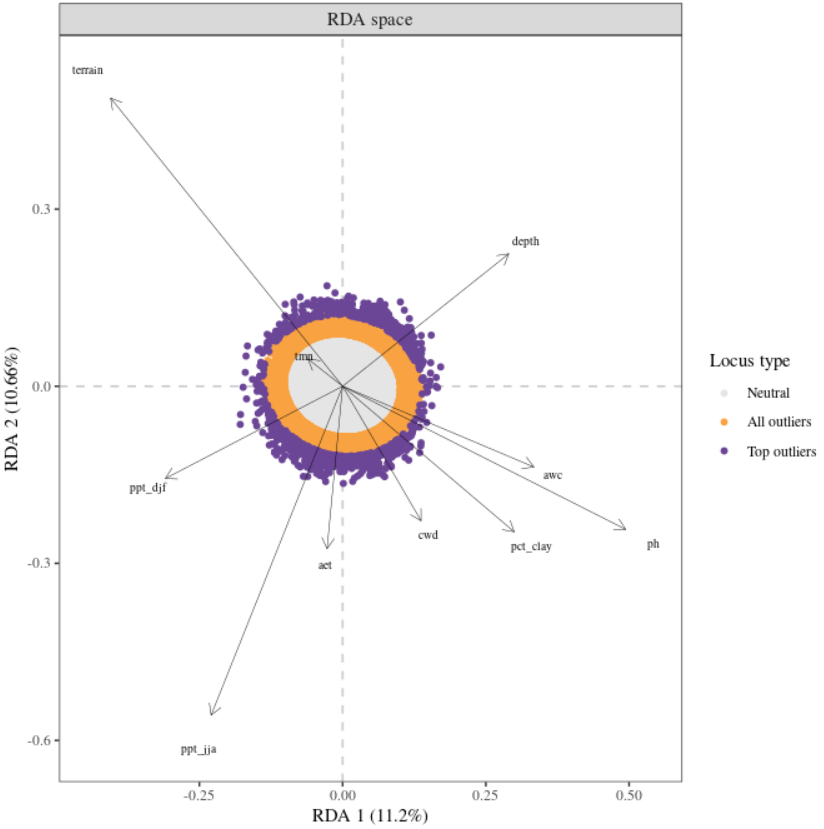
 **Figure S6.** Distribution of *J. californica* genomic variation in RDA space using all loci, with RDA1 accounting for 11.2% of variation and RDA2 accounting for 10.66%. The 10,819 outlier loci above the Bonferroni-corrected significance threshold (α=0.01/n) are colored orange, with the highest 10% of those p-values (1,083 loci) colored purple, while the other 704,456 loci are colored grey. The strength of each environmental variable on variation is represented by the blue loading arrows, with longer arrows representing more importance in explaining variation in the direction they are pointing.

**
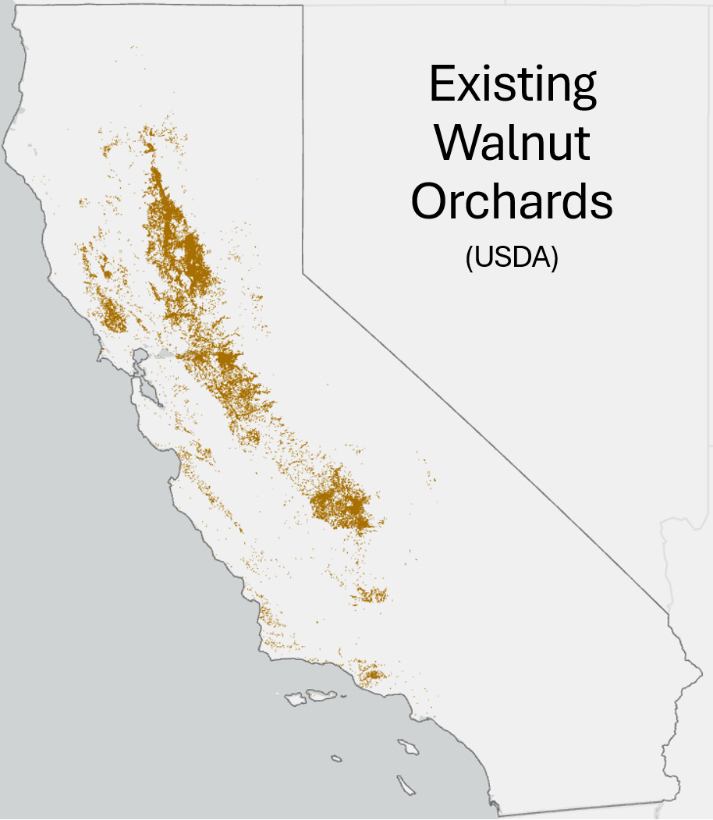
**

**Figure S7.** Map of existing walnut orchards (brown) in California. Extracted from the USDA Cropland Data Layer.

**
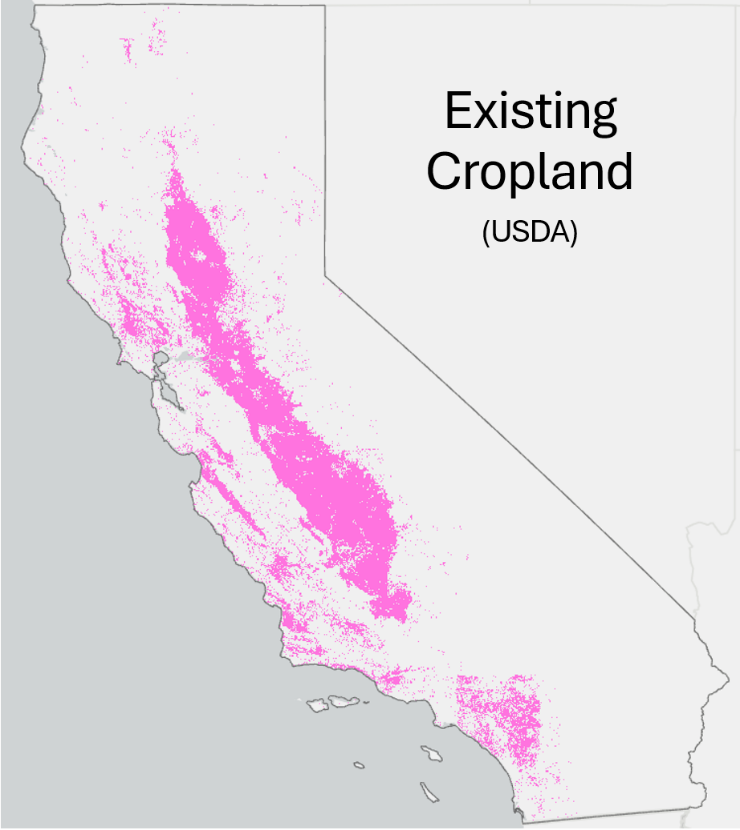
**

**Figure S8.** Map of all existing cropland types (pink) in California. Extracted from the USDA Cropland Data Layer.


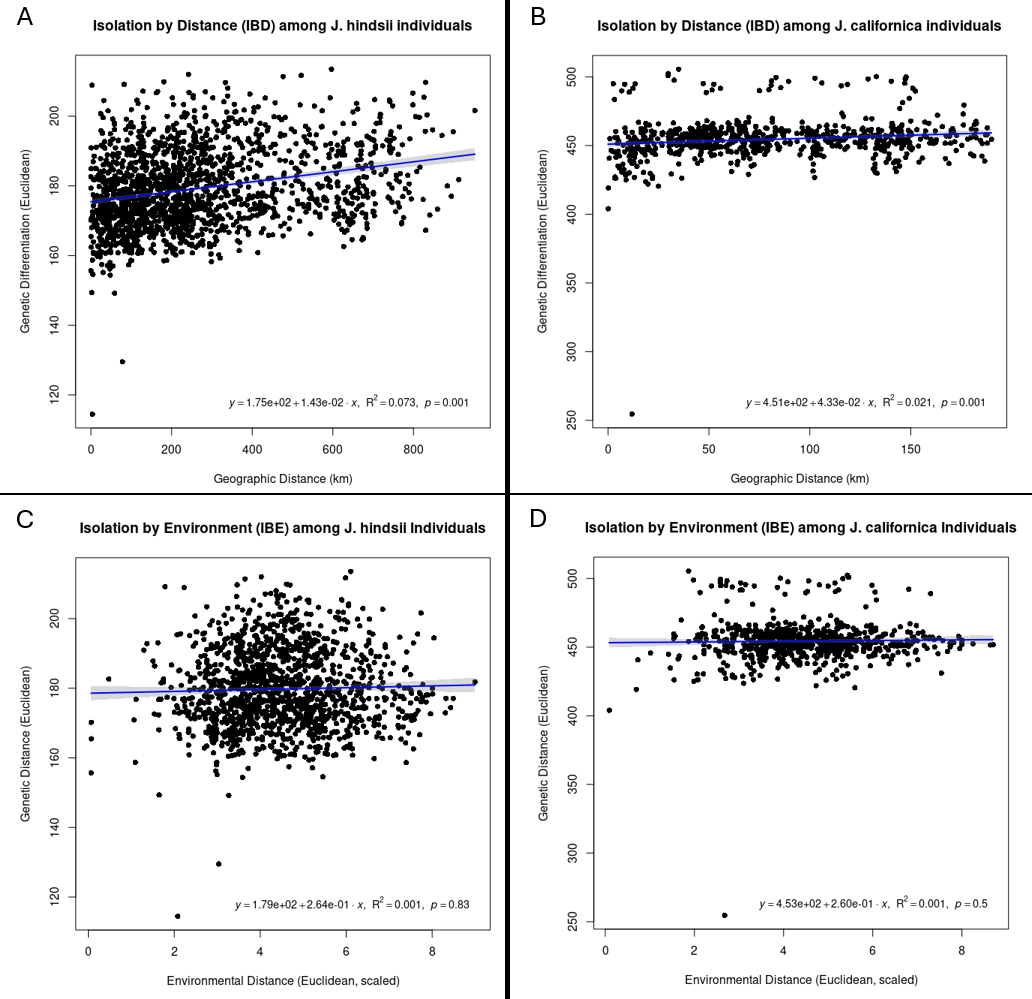
 **Figure S9.** (A and B) Isolation by distance results showing geographic distance in kilometers and genetic distance (as the Euclidean distance between individual genotypes) with each dot representing a pairwise comparison between individuals. (A) For Northern California black walnut (*J. hindsii*), genetic distance has a slight trend (R^2^ = 0.073) with geographic distance, showing minor evidence of isolation by distance (p = 0.001). (B) For Southern California black walnut (*J. californica*), genetic distance has a slight trend (R^2^ = 0.021) with geographic distance, showing minor evidence of isolation by distance (p = 0.001). (C and D) Isolation by environment accounting for geography showing environmental distance (calculated as the Euclidean distances of scaled mean environmental variables at each individual’s location) and genetic distance (calculated as the Euclidean distance between individual genotypes) with each dot representing a pairwise comparison between individuals. (C) For Northern California black walnut, genetic distance has a nonsignificant trend (R^2^ = 0.001, p = 0.81) with environmental distance, showing no evidence of isolation by environment. (D) For Southern California black walnut, genetic distance has a nonsignificant trend (R^2^ = 0.001, p = 0.5) with environmental distance, showing no evidence of isolation by environment.


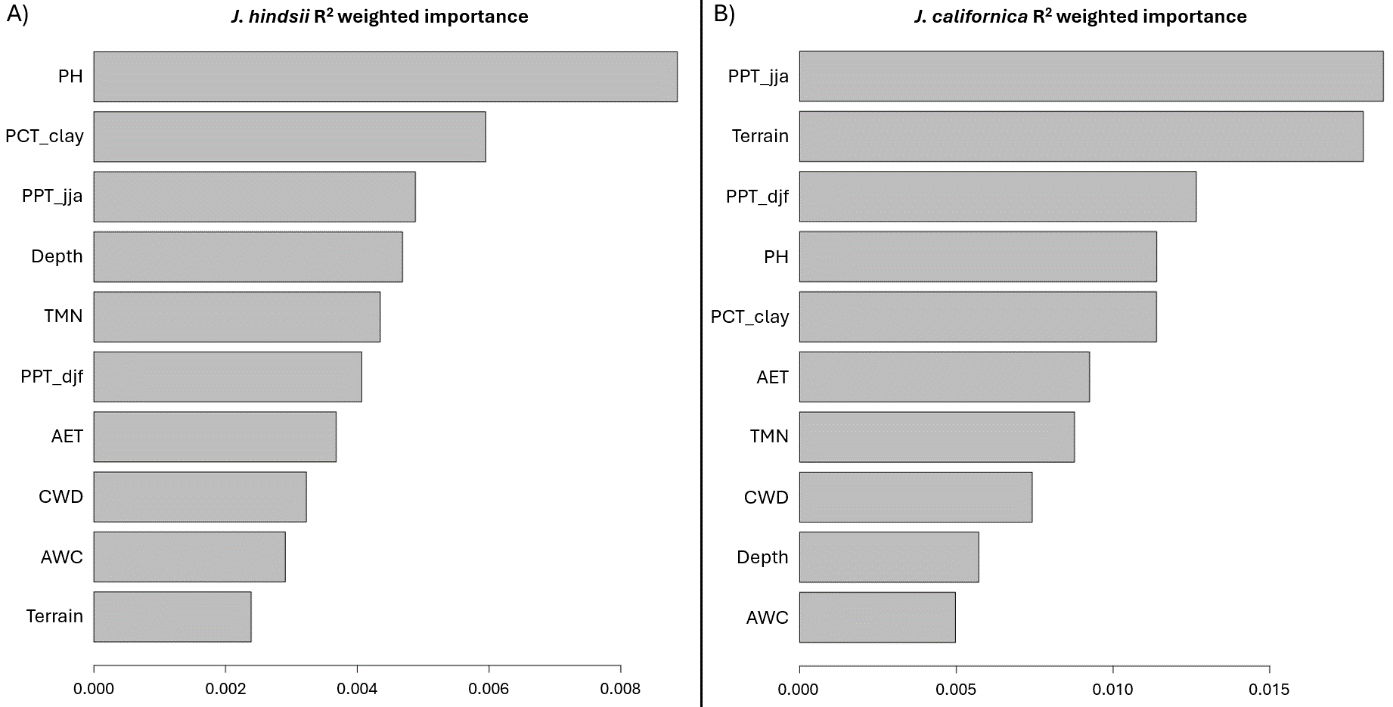


**Figure S10.** Importance of environmental variables in the gradient forest models for wild Northern (A) and Southern (B) California black walnuts. Each graph shows the split importance weighted by the variance explained for each locus. Soil pH is the most important variable in explaining putative adaptive variation in Northern California black walnut, while summer precipitation is the most important for Southern California black walnut.

**
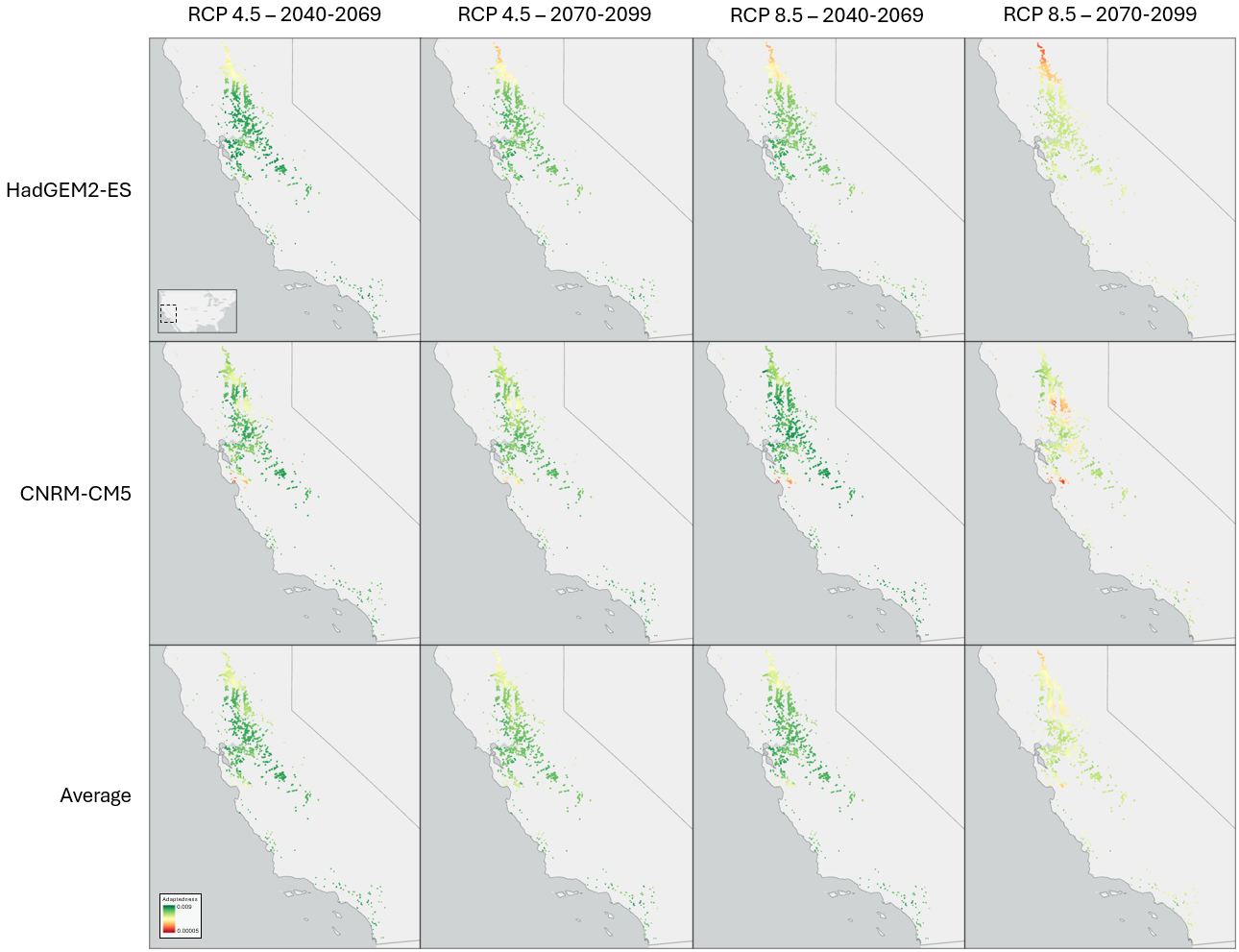
**

**Figure S11.** Climate adaptedness predictions for *J. hindsii* using Gradient Forest for climate models HadGEM2-ES and CNRM-CM5, RCP 4.5 and 8.5, and 30-year average time periods 2040-2069 and 2070-2099. Greener colors indicate higher climate adaptedness values.


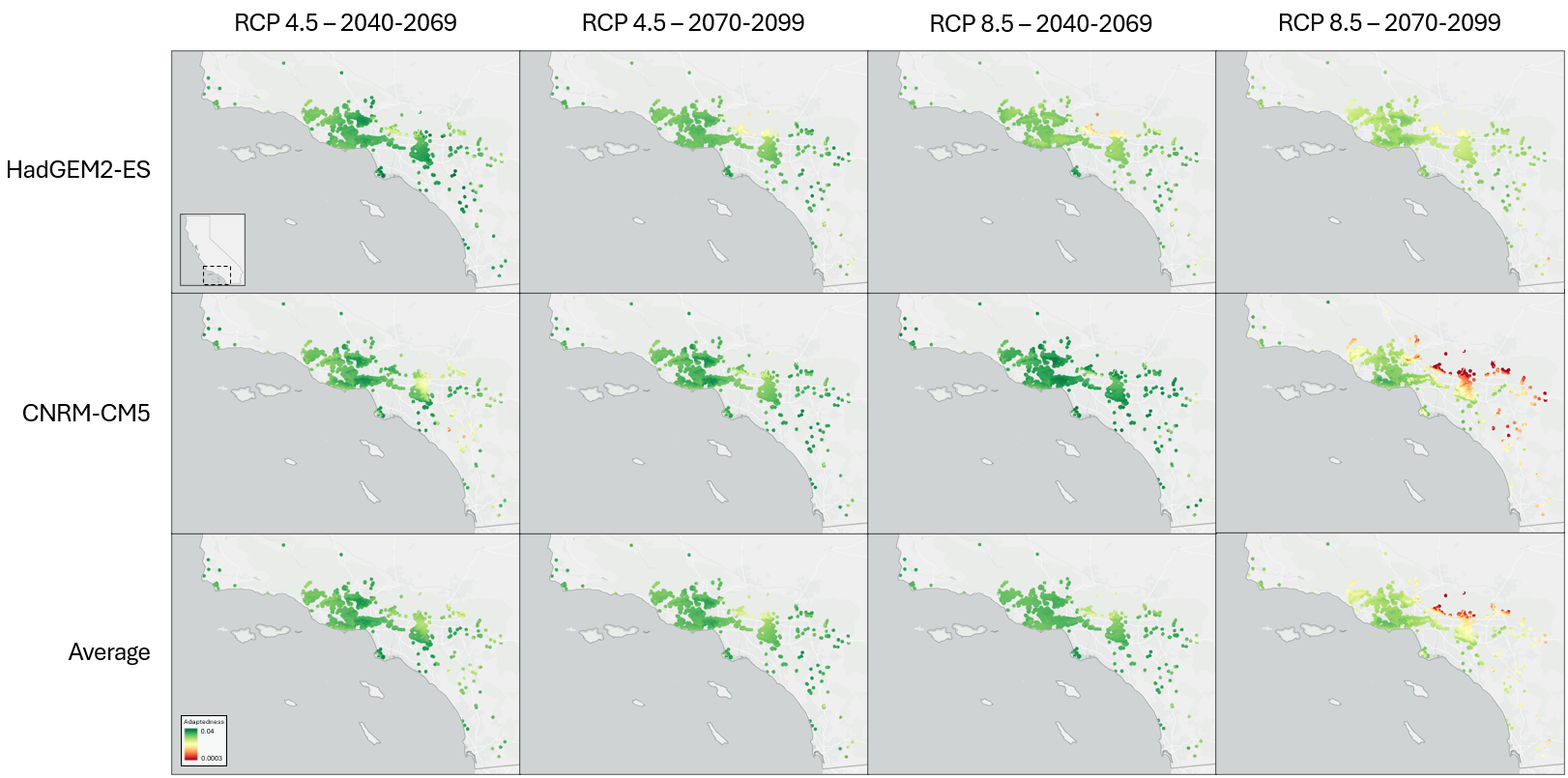
 **Figure S12.** Climate adaptedness predictions for *J. californica* using Gradient Forest for climate models HadGEM2-ES and CNRM-CM5, RCP 4.5 and 8.5, and 30-year average time periods 2040-2069 and 2070-2099. Greener colors indicate higher climate adaptedness values.
